# *Yersinia* Controls Type III Effector Delivery into Host Cells by Modulating Rho Activity

**DOI:** 10.1371/journal.ppat.0040003

**Published:** 2008-01-11

**Authors:** Edison Mejía, James B Bliska, Gloria I Viboud

**Affiliations:** Department of Molecular Genetics and Microbiology, Center for Infectious Diseases, School of Medicine, State University of New York at Stony Brook, Stony Brook, New York, United States of America; Harvard Medical School, United States of America

## Abstract

Yersinia pseudotuberculosis binds to β1 integrin receptors, and uses the type III secretion proteins YopB and YopD to introduce pores and to translocate Yop effectors directly into host cells. Y. pseudotuberculosis lacking effectors that inhibit Rho GTPases, YopE and YopT, have high pore forming activity. Here, we present evidence that Y. pseudotuberculosis selectively modulates Rho activity to induce cellular changes that control pore formation and effector translocation. Inhibition of actin polymerization decreased pore formation and YopE translocation in HeLa cells infected with Y. pseudotuberculosis. Inactivation of Rho, Rac, and Cdc42 by treatment with Clostridium difficile toxin B inhibited pore formation and YopE translocation in infected HeLa cells. Expression of a dominant negative form of Rac did not reduce the uptake of membrane impermeable dyes in HeLa cells infected with a pore forming strain YopEHJT^−^. Similarly, the Rac inhibitor NSC23766 did not decrease pore formation or translocation, although it efficiently hindered Rac-dependent bacterial uptake. In contrast, C. botulinum C3 potently reduced pore formation and translocation, implicating Rho A, B, and/or C in the control of the Yop delivery. An invasin mutant (Y. pseudotuberculosis invD911E) that binds to β1 integrins, but inefficiently transduces signals through the receptors, was defective for YopE translocation. Interfering with the β1 integrin signaling pathway, by inhibiting Src kinase activity, negatively affected YopE translocation. Additionally, Y. pseudotuberculosis infection activated Rho by a mechanism that was dependent on YopB and on high affinity bacteria interaction with β1 integrin receptors. We propose that Rho activation, mediated by signals triggered by the YopB/YopD translocon and from engagement of β1 integrin receptors, stimulates actin polymerization and activates the translocation process, and that once the Yops are translocated, the action of YopE or YopT terminate delivery of Yops and prevents pore formation.

## Author Summary

The type III secretion system (TTSS) is essential for the virulence of a number of Gram-negative human pathogens of enormous clinical significance. The molecular mechanisms by which TTSS effector proteins are translocated into the host cell are not well understood. The work presented here proposes a new model in which the enteropathogen Yersinia pseudotuberculosis manipulates the host cell machinery to control effector translocation. This involves activation of the host cell Rho GTPase by the cooperative action of adhesin-mediated high affinity binding to specific cell receptor molecules known as β1 integrins, and interaction of components of the TTSS with the host cell membrane. This molecular mechanism of controlling TTSS may not be restricted to Y. pseudotuberculosis and might take place during infection of host cells with other pathogens that encode homologues of *Yersinia* TTSS proteins. Our findings provide a good starting point to study the molecular nature of the complex interaction between bacterial pathogens bearing TTSSs and the host cell. Importantly, components that act by modulating the TTSS are potential targets for novel antimicrobials.

## Introduction

A great spectrum of Gram-negative bacteria depends on a specialized secretion mechanism to establish a successful infection in the host. This machinery is known as the type III secretion system (TTSS), and is present in organisms that are pathogenic for animals or plants, as well as in symbiotic bacteria [1]. In pathogenic *Yersinia* species, a TTSS is encoded in a large virulence plasmid, and is required for counteracting innate and adaptive host immune defenses [2]. This is accomplished by injection of six effector proteins (YopE, YopT, YopH, YopJ, YopO, YopM) that target different host cell signaling molecules. This injection mechanism is known as Yop translocation.

Two effectors relevant to this work are YopE and YopT, which target a family of Rho GTPases that control a variety of cellular functions, including regulation of the actin cytoskeleton. In turn, the activity of the Rho GTPases is tightly controlled by a number of regulators. Guanine nucleotide exchange factors (GEFs) induce activation of GTPases by inducing GDP/GTP exchange. GTPase accelerating proteins (GAPs) inactivate Rho GTPases by stimulating GTP hydrolysis. Active Rho proteins are mostly associated with cellular membranes by means of a post-translational lipid modification (prenylation) [3]. YopE inhibits RhoGTPases by acting as a GAP for RhoA, Rac1, or Cdc42 [4,5]. YopT inhibits preferably RhoA, by cleaving the isoprenyl group and removing the GTPase from the membrane [6].

Although the mechanism of translocation is not completely understood, it is thought that effectors are delivered from the bacterial cytoplasm to the outer membrane through a secretion conduit. In turn, this channel is connected to a needle–like structure that transports the effectors directly into the host cell's cytoplasm. Apart from the proteins that form the needle, three translocator proteins (YopB, YopD and LcrV) are required for the delivery of toxins into the host cell. YopB and YopD are thought to form a translocation channel at the plasma membrane [7–9]. Two recent report show that LcrV is located at the tip of the needle [10], and that it may act as an assembly platform for YopB and YopD prior to their insertion in the membrane [11].

Activation upon contact of the bacteria with the host cell is one of the hallmarks of the TTSS. Adhesion of *Yersinia* to host cells is mediated by surface proteins, such as invasin or YadA binding to β1 integrin host cell receptors, or by pH6 antigen interacting with glycosphingolipids [12,13]. High affinity interaction of β1 integrin receptor with invasin, or YadA (via fibronectin), stimulates a signal transduction pathway that involves activation of Src protein tyrosine kinase, tyrosine phosphorylation of focal adhesion proteins, such as FAK and Cas, and downstream activation of Rac1 and PI3-K [12,14,15]. Stimulation of this pathway results in bacterial internalization.

We have previously shown that infection of epithelial cells with Y. pseudotuberculosis lacking YopE, YopT, YopJ and YopH elicits a proinflammatory signaling response that requires YopB but is independent of YopD, suggesting that this signaling event can occur in the absence of a translocation channel [16]. This proinflammatory response, characterized by activation of MAP kinases and NFκB, and production of IL-8, is blocked by the Rho GTPase inhibitory action of YopE, and to a lesser extent YopT [17]. It is therefore possible that YopB elicits activation of a signaling pathway involving Rho GTPases.

Although a translocation channel composed of YopB and YopD is thought to insert into the host cell membrane, the integrity of the plasma membrane remains intact during infection with wild type *Yersinia*. However, infection with *Yersinia* mutant strains that do not produce YopE and YopT results in loss of membrane integrity, a process known as pore formation [7,18]. Interestingly, *yopE,yopT* mutants also induce the polymerization of an actin ring at the site of the interaction with the host cell, but the link between these “actin halos” and pore formation is not known. How YopE or YopT prevent pore formation is not fully understood, and is a controversial issue [19]. We have found that, catalytically inactive forms of YopE or YopT ([18], unpublished data) were not able to prevent pore formation, analyzed by uptake of impermeable dyes (EtdBr) or release of lactate dehydrogenase (LDH). Expression of constitutively active forms of RhoA or Rac1 prior to infection, rescued the pore forming activity of bacteria expressing YopE or YopT [18]. In addition, infection carried out in the presence of actin polymerization inhibitors dramatically reduced pore formation. Based on these results we concluded that insertion of the YopB/D translocation channel results in Rho GTPases activation, actin polymerization, and pore formation [18]. Here, we present evidence that not only pore formation but most importantly, translocation is controlled by Rho activity and actin polymerization. We also found that high affinity interaction between YadA or invasin with β1 integrin receptors is crucial for efficient translocation of Yops. Thus, we hypothesize that YopB/D signaling, in cooperation with β1 integrin signaling, activates Rho to induce changes in the host cell cytoskeleton that control the translocation process.

## Results

### YopB/D-Mediated Pore Formation Is Independent of Caspase-1 Activation

**Figure 1:**
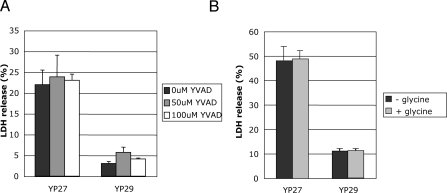
Effect of Caspase-1 Inhibitor or Glycine on YopB/D-Mediated LDH Release HeLa cells were left untreated or treated with 50 μM or 100 μM caspase-1 inhibitor Ac-YVAD-cmk one hour before infection **(A)** or through out the infection with 5mM glycine **(B).** After 3 h infection with a *yopEHJ* mutant (YP27) or *yopEHJB* mutant (YP29), culture supernatants were removed and tested for LDH release using CytoTox 96 assay kit (Promega). The percentage of LDH release was calculated by dividing the amount of LDH release from infected cells by the amount of LDH release from uninfected cells lysed by a freeze-thaw cycle. Error bars represent the standard deviation of the mean values obtained from three infected wells.

Macrophages infected with *Salmonella* or *Shigella* species undergo a caspase-1-dependent form of cell death termed pyroptosis [20]. This death mechanism is proinflammatory, and requires *Yersinia* YopB homologues SipB and IpaB, from *Salmonella* and *Shigella*, respectively. A recent report shows that pyroptosis is caused by caspase-1-dependent pore formation and consequent osmotic lysis [21]. Pore formation is usually determined by the incorporation or release of membrane impermeable dyes, such as EtdBr and BCECF, respectively, by the infected cells [7,8,22]. Because pore formation is followed by osmotic lysis, an indirect method to determine pore formation involves measuring the release of the cytoplasmic enzyme lactate dehydrogenase (LDH) in supernatants of cultured cells [22]. In *Yersinia*-infected macrophages, caspase-1-mediated maturation and release of the proinflammatory cytokine interleukin 1β can be inhibited by YopE and YopT [23]. Because the inhibitory action of YopE and YopT on the Rho GTPases also blocks pore formation [18], we investigated whether YopB/D-mediated cell lysis in HeLa cells is a result of caspase-1 mediated cell death. We used Ac-YVAD-cmk (YVAD), a permeable peptide that specifically inhibits caspase-1, irreversibly. HeLa cells treated for 1 h with 50 μM or 100 μM of YVAD, or control untreated cells, were infected with pore forming strain *yopEHJ* (YP27), and the corresponding pore forming-deficient strain that lacks YopB (*yopEHJB*, YP29). The uptake of the impermeable dye ethidium homodimer-2 (EthD2) and the amount of LDH released in the supernatant of infected cells was tested 3 hours after infection. YVAD did not prevent LDH release (Figure 1A) or penetration of the dye (not shown) in cells infected with YP27. On the other hand, YVAD treatment dramatically inhibited YP27-induced IL-1β production in J774.1A macrophage-like cells (Figure S1), indicating that 100μM YVAD efficiently inhibits caspase-1 mediated processes. These data support the hypothesis that YopB/D-mediated loss of membrane integrity in epithelial cells does not require caspase-1 activation.

*Salmonella*–induced pyroptosis is also inhibited by 5 mM glycine [20]. We investigated if YopB/D-induced loss of membrane integrity could be inhibited by treatment with 5 mM glycine through out the infection. As shown in Figure 1B, glycine had no effect on the amount of LDH released by YP27-infected cells. This result further suggests that in HeLa cells YopB/D-mediated LDH release occurs by a process different from pyroptosis. We therefore consider that, in our experimental system, pore formation is linked to the translocation process.

### Rho GTPase Activation Promotes Pore Formation and Yop Translocation

**Figure 2:**
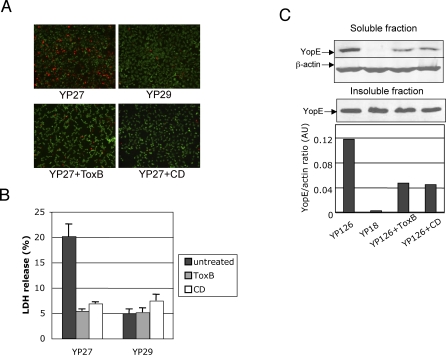
Effect of ToxB or Cytochalasin D on Pore Formation and Yop Translocation HeLa cells were left untreated, exposed to 40ng/ml C. difficile Toxin B (ToxB), or 3.9μM cytochalasin D (CD) for 2 hours prior to infection. Cells on coverslips were infected with *yopEHJ* mutant (YP27) or *yopEHJB* mutant (YP29) for 3 h, and stained with DEAD-LIVE kit, as described in Material and Methods. Cells with disrupted membranes exhibit a red nuclei staining (A). LDH release was determined in the culture supernatants 3h post infection (B). Wild type (YP126) and *yopB* mutant (YP18) were used to infect Hela cells for 2 hours. Triton X-100 cell lysates were centrifuged, and soluble and insoluble fractions (containing translocated Yops and bacterial Yops, respectively) were analyzed by immunoblotting using anti-YopE antibodies. Anti-β actin antibody was used as a loading control of the soluble fraction. Anti-rabbit antibodies conjugated with IR800 or IR680 were used as secondary antibodies, and the infrared signal was detected using the Li-Cor Odyssey infrared scanner. The intensity of each band was calculated using the software provided by the Odyssey IR imaging system, and the YopE/β-actin ratios were plotted on a graph (C).

We have previously found that pore formation is prevented by the catalytic activity of two Rho GTPase-inhibiting effectors, YopE and YopT [18]. To test whether inactivation of small GTPases inhibits pore formation, we incubated cells for 2 h in the presence or absence of 40ng/ml of Clostridium difficile toxin B (ToxB), an ADP-ribosylating protein that powerfully inhibits Rho, Rac and Cdc42. ToxB treatment strongly reduced the uptake of ethidium homodimer-2 (EthD-2) by cells infected with pore forming strain *yopEHJ* (YP27) (Figure 2A). Rho GTPase downregulation by ToxB also inhibited LDH release (Figure 2B). Thus supernatants of YP27-infected cells treated with ToxB released levels of LDH comparable to those of cells infected with the pore-forming-deficient strain *yopEHJB* (YP29). These data suggest that YopB/D-mediated pore formation requires activation of Rho GTPases.

We have previously observed that a catalytically inactive form of YopE (YopER144A) is translocated at higher levels than wild type YopE [4,18]. Aili et al. have also reported this phenomenon recently; they showed that several YopE mutants defective for GAP activity are hypertranslocated [24,25] . Interestingly, Wong and Isberg [26] observed that overexpression of YopT inhibits YopE translocation. Altogether, these observations suggest a possible role of GTPase activation in controlling the translocation process. To study this hypothesis we tested the action of ToxB on YopE translocation using the Triton X-100 solubility assay described in Material and Methods. Pretreatment of HeLa cells with ToxB reduced the amount of YopE translocated by wild type strain YP126 by 60% (Figure 2C). As expected, only background levels of YopE were detected in the soluble fraction of cells infected with the translocation deficient YopB^−^ mutant, YP18. The inhibitory effect of ToxB on pore formation and translocation is not likely to be a consequence of an impairment of the bacteria-host cell interaction, because the number of cell-associated bacteria did not vary with ToxB treatment (Figure S2). This led us to conclude that Yop translocation is strongly influenced by the level of Rho, Rac or Cdc42 activation.

### Inhibition of Actin Polymerization Decreases Yop Translocation

In previous experiments, we have shown that actin polymerization inhibitors, cytochalasin D (CD) and latrunculin B, inhibit pore formation [18]. Here we confirmed the effect of CD on pore formation (Figures 2A and 2B), and determined whether host actin polymerization plays a role in Yop translocation during *Yersinia* infection. As shown in Figure 2C, CD treatment greatly reduced the amount of translocated YopE, this inhibitory effect being comparable to the ToxB treatment. Adhesion assays showed that CD does not affect the number of cell-associated bacteria greatly (not shown). These observations appear to indicate that actin polymerization is not only required for pore formation, as we had shown previously, but it also controls Yop translocation.

### Invasin- or YadA-Mediated Adhesion Promotes Pore Formation and Yop Translocation

Y. pseudotuberculosis internalization into epithelial cells requires a signaling cascade that results from the binding of invasin or YadA to β1 integrin receptors. Bacterial uptake requires small Rho GTPases activation and actin polymerization. Thus internalization, like pore formation and translocation, is inhibited by the GAP activity of YopE, and by treatment with cytochalasin D [4,18]. With this in mind, we investigated whether invasin or YadA-mediated adhesion to β1 integrin receptors is required for efficient pore formation and translocation. We created a *yopEHJ,yadA,inv* mutant strain, designated YP50, and the corresponding YopB-deficient mutant YP51 (Table 1). To provide a means of adhesion, a pAY66 plasmid, constitutively expressing pH6 antigen (Table 1), was inserted into YP50 and YP51. The pH6 antigen is a fimbrial adhesin that can mediate adhesion of *Yersina* to epithelial cells but does not induce bacterial uptake [27]. The defect in internalization of YP50/pAY66 and YP51/pAY66 was confirmed by immunofluorescence (not shown, see below). To corroborate that pH6 ag can substitute invasin or YadA for adherence, we evaluated the binding ability of the YP50/pAY66 strain after one hour infection by immunofluorescence. We found that YP50/pAY66 adhered to HeLa cells at levels similar to *yopEHJ* (YP27) expressing invasin or YadA (not shown).

**Table 1:**
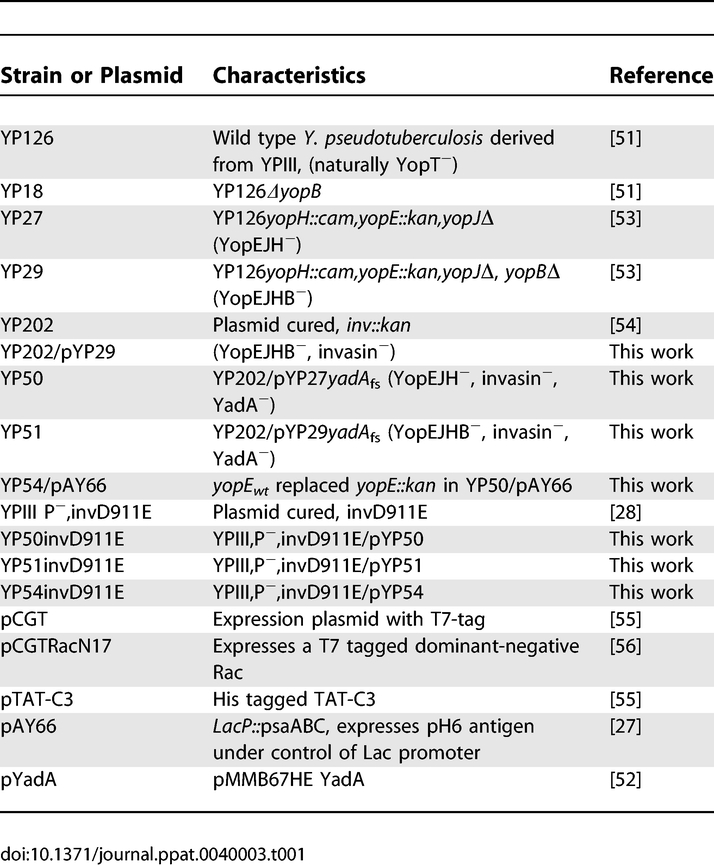
Characteristics of the Strains and Plasmids Used in This Study

**Figure 3:**
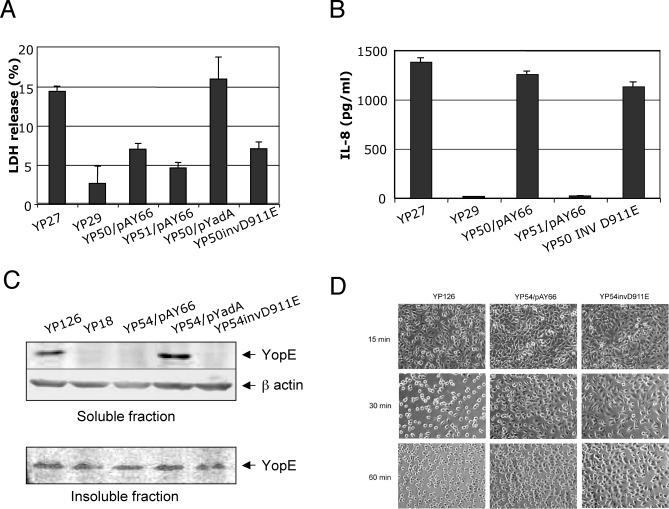
Effect of Invasin and yadA Inactivation on Pore Formation and Translocation HeLa cells were infected with Y. pseudotuberculosis strains *yopEHJ* (YP27), *yopEHJB* (YP29), *yopEHJ,yadA,inv*/p*psaABC* (YP50/pAY66), *yopEHJB,yadA,inv*/p*psaABC* (YP51/pAY66), *yopEHJ,yadA,inv*/pMMB67HE YadA (YP50/pYadA), or *yopEHJ,yadA,invD911E* (YP50invD911E). LDH was determined in the culture supernatants as described in Figure 1 (A). Culture supernatants were collected from triplicate wells 5h post infection and assayed using an IL-8 ELISA (Antigenix America) (B). Y. pseudotuberculosis wild type (YP126), *yopB* mutant (YP18), *yopHJ,yadA,inv*/*psaABC* (YP54/pAY66), *yopHJ,yadA,inv*/pMMB67HE YadA (YP54/pYadA), or *yopHJ,yadA,invD911E* (YP54invD911E) strains were used to infect HeLa cells for 2 hours, and translocated YopE was analyzed by immunoblotting as described in Figure 2. Soluble fractions correspond to translocated YopE, and insoluble fractions correspond to bacteria-associated YopE (C). YopE-mediated cytotoxicity was analyzed by phase contrast microscopy at 15, 30 and 60 min post infection in cells infected with wild type (YP126) strain, YP54/pAY66 or YP54invD911 (D).

YP50/pAY66 strain was compared to the YP27 strain for the ability to induce pore formation. Surprisingly, YP50/pAY66 caused lower levels of LDH release than YP27 (Figure 3A), and was defective for promoting uptake of EthD-2 by infected HeLa cells (not shown). As expected, infection with the corresponding *yopB* mutant YP51/pAY66 resulted in even lower levels of LDH release. Ectopic expression of YadA in the YP50 strain rescued LDH release, indicating that interaction with β1 integrin receptors is critical for pore formation. To rule out that the impairment of the *inv,yadA*, pH6 antigen-expressing mutant to cause pore formation was due to a defective activation of the TTSS, we tested the ability of YP50/pAY66 to induce IL-8 production, NFκB activation, and ERK phosphorylation. We have previously found that the ability to stimulate these pro-inflammatory signals requires YopB but is independent of pore formation [16]. As shown in Figure 3B, after 5 hours infection, IL-8 production was not considerably reduced by the absence of invasin or YadA. Similarly, YopB-dependent activation of NFκB and ERK, measured at 1 hour-post infection, did not require invasin or YadA (Figure S3), suggesting that YopB is able to stimulate cell responses whether adhesion is provided by invasin/YadA or by pH6 antigen. Collectively, these results indicate that interaction of the bacteria with β1 integrin receptors is required to stimulate pore formation.

To investigate whether engagement of β1 integrin receptors is also needed for the translocation process, we tested the ability of a *yadA,inv* mutant to translocate YopE. To this end, we replaced the mutated *yopE* by the wild type *yopE* gene in YP50/pAY66, creating YP54/pAY66 (Table 1). In line with its reduced ability to cause pore formation, the *inv,yadA*, pH6 antigen-expressing mutant translocated undetectable levels of YopE (Figure 3C). Consequent with these findings, YP54/pAY66 induced cell rounding at a much slower rate than the wild type YP126 (Figure 3D, compare YP126 and YP54/pAY66 after 30 min infection). Efficient YopE translocation was restored when YadA was expressed in YP54 (Figure 3C). This suggests that the interaction of Y. pseudotuberculosis with β1 integrin receptors is required for an effective translocation process.

### A Y. pseudotuberculosis Invasin D911E Mutant Is Deficient in Pore Formation and Translocation

As invasin and YadA promote both binding to β1 integrins and stimulation of signaling by this receptor, we used a mutant that is competent for binding to β1 integrins but defective in signaling, to establish which activity was important for pore formation and translocation. A single amino acid substitution, D911E, in the invasin protein retains binding to host cells, but results in low affinity interaction with β1 integrins, poor receptor clustering, and a consequent defect in signaling and internalization [28]. Thus, we assessed the ability of YP50invD911E and YP54invD911E to induce pore formation and to mediate YopE translocation, respectively. Although infection with YP50invD911E resulted in robust IL-8 production (Figure 3B), the levels of LDH release by cells infected with YP50invD911E were as low as those cells infected with the strains that adhere via pH6 antigen (Figure 3A). Similarly, YP54invD911E was impaired in YopE translocation (Figures 3C and 3D). We ruled out that the defect in translocation was a consequence of fewer YP54invD911E bacteria binding to Hela cells. Thus, immunofluorescence analyses after 1-hour infection revealed that YP50invD911E infected cells had a mean of 16.6 associated bacteria/cell, only slightly lower than the invasin-expressing strain (19.7 bacteria/cell, Figure S4A). Moreover, a two-fold increase in the multiplicity of infection of YP54/pAY66 and YP54invD911E did not result in higher levels of YopE translocation (Figure S4B). We conclude that efficient translocation and pore formation involve high affinity binding to β1 integrin receptors.

To examine the binding characteristics of the inv/yadA mutant we performed transmission electron microscopy in thin section of infected HeLa cells. As expected, *yopEHJ* (YP27) bacteria were either internalized, or were in the process of being engulfed, and tightly attached to the host cells (Figure S5A). On the other hand, *yopEHJ,yadA,inv/psaABC* (YP50/pAY66) were almost exclusively extracellular and seemed to bind more loosely (Figure S5B). Adhesion mediated by invD911E differed from that imparted by wild type invasin (Figure S5A and S5C). This suggests that lack of high affinity binding to β1 integrin receptors not only impairs β1 integrin signaling, but might also affect the way the bacteria interacts with the host cell.

### Selective Inhibition of Src Family of Tyrosine Kinases Impairs Effective Yop Translocation

**Figure 4:**
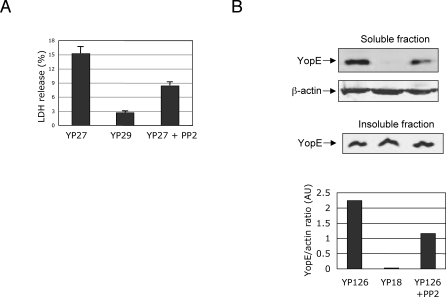
Effect of Src Kinase Inhibitor PP2 on Pore Formation and Yop Translocation HeLa cells were exposed to 10 μM PP2 in DMSO or to DMSO alone one hour prior to infection. HeLa cells infected with YP27 (*yopEHJ)* or YP29 (*yopEHJB)* were assessed for pore formation as indicated for Figure 1 A. Wild type (YP126) and *yopB* mutant (YP18) were used to infect treated HeLa cells for 2 hours. YopE translocation was determined as described in Figure legend 2B.

Stimulation of signaling through β1 integrins receptor by invasin and YadA involves tyrosine phosphorylation of a series of signaling proteins. Src is a key signal-transducing protein kinase in the β1 signaling pathway leading to internalization. To determine if Src activation plays a role in Yop translocation, we tested the effect of a selective inhibitor of Src family kinases, PP2, on infected cells. Pre-treatment of cells for 1 hour with 10μM PP2 efficiently inhibited β1 integrin signaling pathway leading to bacterial internalization without decreasing bacterial adherence (not shown). Interestingly, pore formation and YopE translocation were also impaired in PP2-treated cells (Figures 4A and B). These data indicate that Src activation stimulates translocation, and point toward a role of β1 integrin signaling in the Yop translocation.

### Internalization Is Not Required for Pore Formation or Translocation

**Figure 5:**
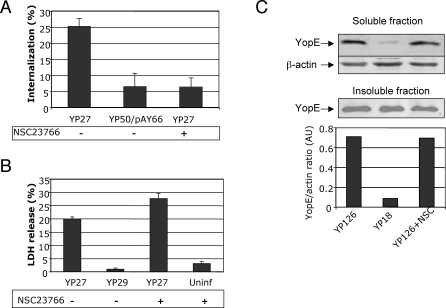
Rac Inactivation Inhibits Bacterial Uptake but Not Pore Formation or Translocation Hela cells were treated for 6h with Rac inhibitor NSC23766 (100μM) in 5% serum-DMEM, or with 5% serum-DMEM alone. NSC23766-treated and untreated cells were infected with YP27 (*yopEHJ)* or the uptake-deficient strain YP50/pAY66 (*yopEHJ,yadA,inv*/*psaABC*). The percentage of internalized bacteria was assessed one hour after infection by double staining immunofluorescence, as described in Material and Methods (A). LDH released by uninfected cells or by cells infected with YP27 or YP29 (*yopEHJB*), in the presence or absence of the Rac inhibitor, was assessed as described in Figure 1 (B). The amount of translocated YopE in the cell lysate of cells infected with wild type (YP126) or *yopB* (YP18) was analyzed by immunoblotting as described Figure 2 (C).

Invasin triggered-Rac1 signaling pathways downstream of Tyr phosphorylation are essential for *Yersinia* uptake [15]. We made use of a specific Rac1 inhibitor to determine whether β1 integrin–mediated internalization was required for efficient pore formation and translocation. NSC23766 is a small chemical compound reported to specifically block the binding between Rac1 and its exclusive GEFs [29]. We tested the effect of the Rac1 inhibitor by pre-treating HeLa cells for 6h with 100μM of NSC23766 in DMEM with 5% serum. As expected, bacterial uptake was impaired by treatment with the Rac inhibitor, with the number of *yopEHJ* (YP27) bacteria internalized by NSC23766-treated cells being comparable to that of the uptake-deficient *yopEHJ,yadA,inv* (YP50/pAY66) strain (Figure 5A). NSC23766 treatment was also found to inhibit formation of phagosomes, as the number of actin cups was reduced more than 5 fold in the presence of the inhibitor (Figure S6). We further excluded any effect of NSC23766 treatment on the number of cell-associated bacteria by immunofluorescence (not shown). Transmission electron microscopy of thin sections also confirmed that NSC23766 inhibited bacterial uptake by, but not association to HeLa cells (Figure S5D). Importantly, treatment with NSC23766 did not reduce pore formation or Yop translocation (Figures 5B and 5C, respectively). These results indicate that bacterial internalization is not required for pore formation or translocation.

To validate our findings using the Rac1 inhibitor, we expressed a dominant negative form of Rac1 in Hela cells. We transfected cells with a eukaryotic expression plasmid coding for a T7 tagged-RacN17 (pCGTRacN17) and we evaluated whether pore formation was impaired in transfected cells. Overexpression of Rac1N17 (green cells) did not prevent pore formation as shown by the uptake of the impermeable dye EthD-2 (Figures S7A and S7B). Altogether, these data provide evidence indicating that neither bacterial internalization, nor Rac1 activation, play a major role in the processes that govern pore formation and Yop translocation.

### Rho Inhibitor C3 Blocks Pore Formation and Inhibits Yop Translocation

**Figure 6:**
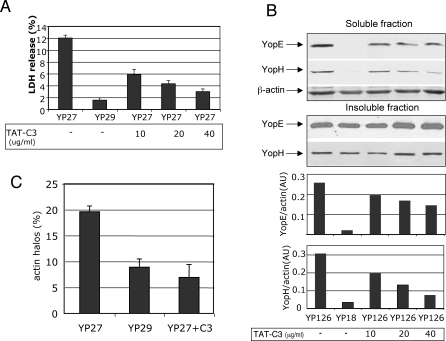
TAT-C3 Treatment Inhibits Pore Formation, Actin Halos, and Translocation in HeLa Cells Hela cells were treated for 4h with 10μg/ml, 20μg/ml, or 40μg/ml TAT-C3 in serum-free DMEM or with serum-free DMEM alone. Cells were infected with *yopEHJ* (YP27) or *yopEHJB* (YP29), in the presence or absence TAT-C3, and LDH released was tested after 3 hours, as described in Figure 1 (A). YopE and YopH translocation into Hela cells infected with wild type (YP126) or translocation-deficient *yopB* (YP18) strain, in the presence and absence of TAT-C3, was analyzed by immunoblotting, as described in Figure 2 (B). Cells seeded on coverslips were treated with 40μg/ml TAT-C3, or left untreated, and infected with *yopEHJ* (YP27) or *yopEHJB* (YP29). After 10 min infection cells were washed and fixed, and subjected to immunofluorescence. Actin was visualized by staining with Rhodamine–phalloidin. Images were acquired by confocal microscopy. Results were expressed as the percentage of bacteria inducing a halo of actin polymerization. A minimum of 250 bacteria was counted (C).

C3 is an ADP-ribosylating protein of Clostridium botulinum that specifically inhibits Rho A, B and C. A recombinant cell-permeable form of C3 toxin (TAT-C3) was produced in E. coli and purified as described in Material and Methods. Four hours before infection, HeLa cells were treated with 10, 20, and 40μg/ml of TAT-C3 in serum-free medium, or with serum-free medium alone. C3 has been previously shown to increase Y. pseudotuberculosis uptake in COS-1 cells [30]; in our experimental model, pretreatment of cells with 20μg/ml TAT-C3 did not affect bacterial adhesion or internalization considerably (Figures S8A and S8B, respectively). Interestingly, TAT-C3 treatment of cells infected with the pore forming strain *yopEHJ* (YP27) inhibited LDH release in a dose-dependent manner (Figure 6A). The effect of Rho inhibition on translocation was also substantial (Figure 6B). In various experiments, treatment with different batches of purified TAT-C3 (40μg/ml) reduced YopE delivery into wild type-infected cells, by 40% to 75 %. Similar results were obtained when we tested the effect of C3 treatment on YopH translocation (Figure 6B), indicating that the requirement of Rho for translocation is not a phenomenon restricted to YopE delivery.

To test whether actin polymerization required for pore formation and translocation was dependent on Rho, we analyzed the effect of C3 on the induction of actin polymerization around the bacteria [18]. We found that the number of YopB-dependent actin halos was considerably reduced in the presence of C3 (Figure 6C).

### Rho Is Activated by a Mechanism That Requires YopB and Invasin/YadA-Mediated Signaling

**Figure 7:**
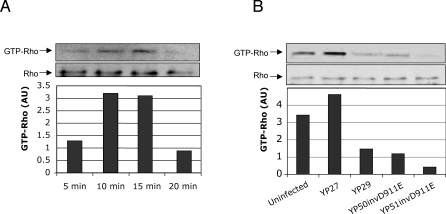
Rho Activation Requires YopB and High Affinity Interaction of the Bacteria with β1 Integrin HeLa cells grown in 10cm diameter dishes were infected with *yopEHJ* (YP27) for 5, 10, 15, and 20 min. GTP-bound active Rho was pulled-down from cell lysates with a GST-fusion protein harboring the Rho binding domain of rhotekin. The precipitates were subjected to immunobloting using an anti-Rho monoclonal antibody. The amount of total Rho was determined in a 20μl aliquot (approx. 3%) of the cell lysates. Results were expressed in arbitrary units (AU) as the ratio between pulled-down GTP-Rho and total Rho (A). HeLa cells were left uninfected or were infected with *yopEHJ* (YP27), *yopEHJB* (YP29), *yopEHJ,yadA,invD911E* (YP50invD911E), and *yopEHJB,yadA,invD911E* (YP51invD911E) for 15min. The amount of GTP-Rho in each of the lysates was determined as described above (B).

To determine whether Rho is activated by infection with *Y. pseudotuberculosis,* we infected HeLa cells with strain *yopEHJ* (YP27) for 5, 10, 15 and 20 min and we analyzed the amount of active Rho (GTP-Rho) in the cell lysates by a GTP-Rho pull-down assay, as described in Material and Methods. A peak of Rho activation was detected between 10 and 15 min after infection (Figure 7A). A 15 min infection period was selected to test the levels of GTP-Rho induced by infection with *yopEHJ* (YP27), *yopEHJB* (YP29), *yopEHJ,yadA,invD911E* (YP50/ invD911E), and *yopEHJB,yadA,invD911E* (YP51/invD911E). Compared to YP27-infected cells, cells infected with YP29 have reduced amounts of GTP-Rho, indicating that Rho activation is YopB-dependent (Figure 7B). Low affinity interaction with β1 integrin receptors by infection with YP50/ invD911E cause a reduced activation of Rho. However, YopB-independent Rho activation in YP29-infected cell lysates was greater than that of cells infected with YP51invD911E. This small difference, attributed to wild type invasin or YadA interacting with β1 integrin receptors, was consistent in three independent experiments. Overall, these experiments lead us to conclude that Y. pseudotuberculosis activates Rho by a process that involves YopB and high affinity interaction with β1 integrin receptors.

## Discussion

The TTSS-mediated translocation of bacterial effectors into host cells is an intricate mechanism that, although extensively studied, has not been completely unraveled [31]. Here we have found that Y. pseudotuberculosis engages the small GTPase Rho to control the delivery of effectors to the host cell. Activation of this signaling pathway is mediated by the YopB/YopD translocon in cooperation with the high affinity binding of invasin or YadA to β-1 integrins.

It has been put forward that pore formation and translocation of effector Yops into the host cells are not related processes [19,32]. Pore formation has been recently implicated in mediating a caspase-1 dependent type of cell death in *Salmonella*-infected macrophages [21]. Shin and Cornelis [33] have recently reported that insertion of translocation pores in macrophages infected with a multi-effector mutant of Y. enterocolitica triggers activation of caspase-1. Here we ruled out that in our infection system, YopB/YopD-mediated pore formation induces caspase-1 dependent cell death. Thus, amounts of a specific caspase-1 inhibitor large enough to block IL-1 β production in macrophages, does not prevent LDH release in Hela cells. Also, glycine treatment that efficiently prevented cell lysis in *Salmonella* infected macrophages failed to inhibit LDH release in *Yersinia*-infected HeLa cells. Based on these findings, we sustain that in our experimental system pore formation-induced LDH release is related to the process of Yop translocation.

Both pore formation and translocation require activation of small Rho GTPases, as glucosylation of Rho, Rac and Cdc42 by C. difficile toxin ToxB potently inhibits the two processes. We found that Rac activation is not likely to be involved in pore formation or translocation. Thus over-expression of a dominant negative form of Rac does not prevent uptake of membrane impermeable dyes in cells infected with the pore forming strain. In line with these results, a specific Rac inhibitor, NSC23766, that efficiently blocks Rac-mediated internalization, does not inhibit pore formation or translocation. On the other hand, we found that signaling downstream of Rho is essential for the control of Yops delivery. Treatment with *C. botulinum* C3 toxin, that converts endogenous Rho A, B and C into dominant negative forms [3], potently down-regulates pore formation and translocation without affecting bacterial adhesion or internalization considerably.

The type of host cell processes that Rho proteins regulate to promote translocation and pore formation most likely involves actin cytoskeleton rearrangements. Thus treatment with 2μg/ml actin polymerization inhibitor CD blocks pore formation [18] and decreases the level of YopE translocation by more than 60%. In early studies aim at demonstrating that Yop translocation is mediated by extracellular bacteria, Sory et al studied the effect of 5μg/ml CD treatment on the delivery of Yop-cyclase fusion proteins by Y. enterocolitica into murine macrophages [34]. Compared to the dramatic effect on bacterial uptake (2000 fold inhibition), the authors suggest that Yop translocation was not sensitive to the action of CD. However, their results show that CD treatment decreased YopE-cyclase translocation by 32% and YopH-cyclase by 52%. Using 10 times less CD (0.5μg/ml for 30 min) and using a strain of *Salmonella* ectopically expressing YopE, Rosqvist *et al* reported that Yop translocation into HeLa cells was notably decreased [35]. The authors also reported that the same was observed when YopE was delivered by Y. pseudotuberculosis. Interestingly, our findings strongly suggest that actin polymerization required for pore formation and translocation is dependent on Rho, as inhibition of Rho A, B and/or C results in a decrease of the number of actin halos.

Adhesion of bacteria to host cell is crucial for the activation of the TTSS. In Y. pseudotuberculosis two main adhesins, invasin and YadA, mediate tight binding to host cells by interaction with β1 integrin receptors. Here we show that in an *inv/yadA* mutant, constitutive expression of the pH6 antigen confers good adhesion properties to host cells. In spite of that, we found that such mutants are defective in pore formation and Yop translocation, suggesting that interaction with β1 integrin receptors is essential for the two processes. Mota *et al.* have shown that a minimal needle length is required for efficient functioning of the *Yersinia* injectisome, and that this length correlates with the length of the YadA protein [36]. We considered that the attachment imparted by pH6 antigen in the absence of invasin and YadA, might not provide that critical length. Our data suggest that this is not likely to be the case in our experimental system. First, a Y. pseudotuberculosis strain expressing pH6 antigen is able to stimulate a YopB-dependent proinflammatory response, including activation of NFκB and ERK, and production of IL-8. Second, a single amino acid substitution in invasin (invD911E), that is not expected to change its length, failed to mediate efficient Yop translocation. This mutant promotes adhesion without inducing receptor clustering and subsequent β1 integrin-mediated signal transduction. Altogether, these results suggest efficient translocation requires high affinity binding of β1 integrin receptors and subsequent activation of signaling. It is still conceivable that, independent of integrin signaling, tight bacterial adhesion mediated by high affinity interaction with β1 receptors preconditions effective translocation. The fact that interfering with β1 integrin signaling by the action of a Src inhibitor impairs efficient translocation, would argue against that idea. Still, we cannot discard the possibility that Src activity might also be required for YopB/D-dependent Rho activation.

We predict that upon integrin clustering, RhoA could be recruited and generate a signal that polymerizes actin. It is well documented that invasin engagement of β1 integrin receptors triggers Rac1-mediated signals that induce bacterial internalization into epithelial cells [15]. This Rac1-mediated mechanism involves Arp2/3, PIP 4,5 and capping-proteins [30]. Results from our GTP-Rho pull down assays suggest that bacteria producing invasin and YadA (YP29) can also mediate Rho activation in a YopB-independent manner. There are further evidences in the literature that engagement of β1 integrin receptors can stimulate RhoA activation. Wong and Isberg have shown that RhoA is recruited at the nascent phagosome in Cos1 cells infected with a *yopE yopT* mutant of *Y pseudotuberculosis* [26]. Werner et al have reported that interaction of invasin-coated beads with α5β1 integrin in synovial fibroblast results in beads uptake by a process that is RhoA-dependent [37]. Also, activation of RhoA by engagement of α5β1 integrins by Ipa invasins has been implicated in the internalization of *Shigella* to HeLa cells [38,39]. Alternatively, β1 integrin may indirectly facilitate Rho activation by a focal adhesion kinase (FAK) -dependent pathway. Such a mechanism of Rho activation has been described for the regulation of microtubules stabilization at the leading edge of mouse fibroblasts [40], and involves targeting of Rho to GM1-rich domains in the plasma membrane, where it can interact with downstream effectors.

**Figure 8:**
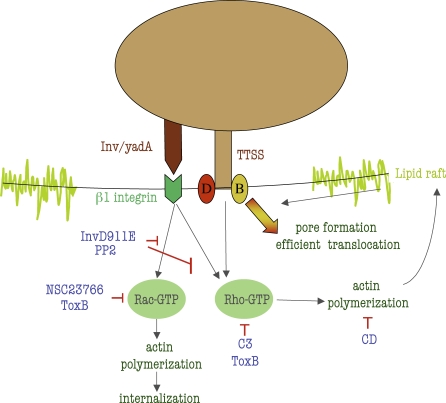
Model for the Requirement of Rho Activation and Actin Polymerization for Pore Formation and Efficient Translocation Upon binding of invasin or YadA to β1 integrin receptor, TTSS is activated, and YopB/D insert in the host cell plasma membrane (in cholesterol-rich domains present in the lipid rafts). YadA/invasin-mediated high affinity binding to β1 integrin receptor activates Rac, and Rho. Membrane-associated YopB/D, stimulates signaling that cooperates with β1 integrins to fully activate Rho. Actin polymerization, resulting from Rho activation, presumably induces lipid raft clustering at the site of the bacterial contact. More injectisomes can then interact with lipid rafts, and more effector Yops are translocated. As soon as enough Yops are translocated, the process is reverted by the inhibitory action of YopE and YopT on the Rho GTPases. Depicted are the inhibitory action on pore formation and translocation of an invasin mutant that binds to β1 integrin with low affinity (InvD911E), the Src inhibitor PP2, the RhoGTPases pan inhibitor ToxB, the specific Rho inhibitor C3, the specific Rac inhibitor NSC23766, and the actin polymerization inhibitor cytochalasin D.

We envision a model in which high affinity binding to β1 integrin receptors, in addition to stimulating Rac activation, triggers Rho activation (Figure 8). Subsequently, YopB/D insertion into the plasma membrane stimulates increased Rho activation, and the cooperative activation of Rho stimulates Yop translocation. A central question is how Rho activation regulates Yop translocation. We hypothesize that Rho signaling induces changes in the host cell, such as actin polymerization, that are required for an efficient translocation process. One possibility is that, cell molecules present in specialized membrane microdomains, such as lipid rafts, are required for efficient translocation. These membrane microdomains would be recruited at the site of bacteria-host cell contact, as a result of Rho GTPases activation and actin polymerization. More injectisomes could then interact with lipid rafts at the site of bacteria, and more effector Yops would be translocated. Once proper amounts of Yops are delivered into the host cell, the process would be shut down to avoid further cell damage caused by excessive signaling. We based our hypothesis, in part, on the fact that *Salmonella* and *Shigella*-YopB homologues bind to cholesterol [41], and that lipid raft are required for translocation in *Salmonella*, *Shigella* and EPEC [41]. Interestingly, actin polymerization and Rho GTPases activation have been shown to be involved in lipid raft clustering in B cells [42], T cells [43] and NK cells [44] .

Why is Rho-dependent, but not Rac-dependent, actin polymerization required for translocation? Rho GTPases transmit signals that control the formation of distinct cytoskeletal structures through the interaction with different nucleating machineries. Cdc42 and Rac mediate nucleation of branched actin filaments through the Arp2/3 protein complex, leading to lamellipodia formation. On the other hand, Rho proteins stimulate unbranched actin filaments formation, such as those in stress fibers, via interaction with formins. It could be speculated that only F-actin structures generated by formins are important for translocation. The effect of the expression of dominant negative mutants of the formin mDia1 on translocation will be investigated in future studies.

Findings from two studies that investigate translocation of TTSS effector proteins by *Salmonella* and *Shigella* in real time [45,46] indicate that effector translocation occurs right after host cell contact, with a half maximal rate of about 4 min. In our experimental model we detect the strongest Rho activation after 10 to 15 min infection with a *YopEHJ* bacteria. This is probably due to accumulation of GTP-Rho in the absence of the Rho inhibitors YopE and YopT. The decrease in the levels of GTP-Rho after 15 min is presumably due by the action of endogenous GAPs. We envision that during infection with wild type bacteria, the kinetics of Rho activation would be much faster. Translocation of *Salmonella* SipA and SopE, and *Shigella* IpaC were found to follow a linear kinetic [45,46]. Interestingly, however, slopes of IpaB secretion kinetics curves seemed to vary at different time points, suggesting that the speed of injection changes during the course of the translocation process resembling a slow-fast-slow type of mechanism. This type of translocation kinetic is what we would expect in our model.

How does our model fit with the mechanism of Yop translocation in Y. pestis? Although closely related to Y. pseudotuberculosis, Y. pestis lacks invasin and YadA. Unless *Y. pestis* has yet-unidentified adhesins that interact with β1 integrin receptors, we envision that the bacteria would activate Rho only by the stimulus elicited by YopB/D. In this situation, Rho activation would be limited, and therefore, one should expect that Y. pestis would be less effective for Yop translocation. A recent report suggests that, in macrophages, Y. pestis translocate less YopJ than a Y. enterocolitica strain expressing invasin and YadA [47]. However, in this report the authors suggest that this is most likely due to a difference between the YopJ protein from the two *Yersinia* species. We have preliminary results suggesting that Y. pestis deliver much less YopE in HeLa cells than Y. pseudotuberculosis.

It has been proposed that, because bacterial effectors are directly injected within cell cytosol, the TTSS does not need to trigger signals through cell surface receptor [48]. Our data suggest that, although not essential, signal stimulated by engagement of β1 integrin receptors greatly enhances Yop translocation.

## Materials and Methods

### Bacterial strains.

The wild-type serogroup III Y. pseudotuberculosis strain YP126 [49], and the mutants derived thereof are shown in Table 1. YP126 and its derivatives carry a naturally occurring deletion in virulence plasmid that inactivates the *yopT* gene and are thus devoid of YopT activity [50].

### Strain construction.

YP202/YP29 (*yopEHJB,inv*) was constructed by inserting the virulence plasmid of YP29 into a plasmid cured, *inv::kan* strain (YP202, Table 1). To create YP50 (*yopEHJ,yadA,inv*) and the corresponding YopB-deficient mutant (YP51), the wild type *yadA* gene in YP202/pYP27 (*yopEHJ,inv*) and in YP202/pYP29 (*yopEHJB,inv*), respectively, was replaced by *yadA* containing a frame shift deletion (*yadAfs*), as follows. *yadAfs* was constructed by amplifying *yadA* with primer YadA F1 (5′-CCC GGG TTT GTA GTG GGC TGA CTC CGA C-3′) and B1 (5′'-GGC TGA ACT GGC TAA ACC TTT G-3′). The *yadA* DNA fragment was subsequently blunt-cloned into pETBlue (Novagen). QuikChange Site-Directed mutagenesis (Stratagene) was used to create the frame-shift and generate a SphI restriction site using primers F2 (5′-CA CAA GGT CCA GAA AAA AAA GAG CAT GCA TTA GCA GAA GCA ATA C-3′), and B2 (5′-GTA TTG CTT CTG CTA ATG CAT GCT CTT TTT TTT CTG GAC CTT GTG-3′). Plasmid pETBlue-*yadA_fs_* was digested with XmaI and subcloned into the suicide plasmid pSB890 containing *sacB* and Tet^R^ genes [51]. pSB890*yadA_fs_* was then introduced into S17-λpir and conjugated into Cam^R^ YP202/pYV27 and YP202/pYV29. Tet^R^ Cam^R^ colonies were grown for several generations in the absence of Tet and were selected against the *sacB* on LB-5% sucrose. Sucrose^R^, Cam^R^ and Tet^S^ colonies were screened for *yadA_fs_* by PCR using primers YadA F1 and B1, followed by SphI-digestion of the amplified *yadA* fragment. A plasmid constitutively expressing pH6 antigen fimbriae, pAY66 (*LacP::*psaABC, Table1), a gift from R. Isberg (Tufts University), was inserted into YP50 and YP51 by electroporation. To create YP54/pAY66 (*yopHJ,inv,yadA*/psaABC), we replaced *yopE::kan* in YP50/pAY66 by wild type *yopE*, by allelic exchange using suicide plasmid pSB890YopE, essentially as described above. To construct YP50invD911E (*yopEHJ,yadA,invD911E*) and YP51invD911E (*yopEHJB,yadA,invD911E*), the virulence plasmid from YP50 and YP51 were introduced into YPIII P^−^ invD911E (Table 1, gift from R. Isberg ) by electroporation. To create YP54invD911E, we replaced *yopE::kan* in YP50invD911E by wild type *yopE*, by allelic exchange using pSB890yopE as described above. Plasmid pMMB67HEYadA (pYadA) [52], was inserted into YP50 and YP54 by electroporation.

### Cell culture and infection conditions.

HeLa cells were cultured as previously described [16]. For experiments carried out in the presence of inhibitors, cells were pre-incubated with 50–100 μM Ac-YVAD-cmk (Calbiochem), 5mM Glycine (Roche), 40ng/ml Clostridium difficile ToxB (Calbiochem), 3.9 μM (2μg/ml) cytochalasin D (Sigma), 10 μM PP2 (Sigma), 100 μM NSC23766 (Calbiochem), 10, 20, or 40 μg/ml TAT-C3. Bacteria used for infections were grown in Luria-Bertani (LB) broth either under conditions that stimulate (low Ca^2+^ at 37 °C) or repress (high Ca^2+^ at 28 °C) Yop expression [4,51], at a multiplicity of infection of 50 to 100. The plates containing the infected cells were centrifuged for 5 min at 700 rpm and incubated at 37 °C with 5% CO_2_ for different periods of time to allow bacterial-host cell interaction.

###  Uptake of impermeable dye ethidium homodimer-2.

Cells cultured in 24-well plates with coverslips were infected for 3 h with bacteria grown under low calcium conditions. A green fluorescent membrane-permeable nucleic acid stain (SYTO10) and a red membrane-impermeable nucleic acid dye that label only cells with compromised membranes, ethidium homodimer-2 (EthD-2) were provided in the DEAD-LIVE kit (Invitrogen). After washing, a mixture of the two dyes was added to the wells and incubated in the darkness for 15 min at room temperature. Cells were then washed and fixed with 2% paraformaldheyde in PBS. Coverslips were mounted with 8 μl of ProLong mounting medium (Molecular Probes) and slides were then examined by immunofluorescence microscopy.

### LDH assay.

Samples of culture media from wells containing infected cells were collected 3 h post infection. Levels of LDH were assayed using the CytoTox 96 assay kit (Promega) as previously described [16].

### Yop translocation assay**.**

HeLa cells cultured in 6 cm^2^ dishes were infected with bacteria grown at high Ca^2+^ conditions. Infected cells were lysed with 0.2 ml of cold 1% Triton X-100 buffer as described [4]. Soluble and insoluble fractions were subjected to immunoblotting using an affinity purified polyclonal anti-YopE and anti YopH antibodies, as described previously [4]. Anti-β actin antibody was used as a loading control. Anti-rabbit antibodies conjugated with IR800 or IR680 were used as secondary antibodies, and the infrared signal was detected using an infrared imaging system (Odyssey, LI-COR). Quantification of a fluorescent signal is more accurate than that generated by chemiluminescence because its intensity is not time-dependent. The bands intensities were calculated using the software provided by the Odyssey system, and the values were expressed as the YopE/β-actin ratio and plotted on a graph.

### IL-8 assay.

Supernatants of infected HeLa cells were assayed for IL-8 production by ELISA (Antigenix America) five h after infection, as previously described [16]. Values obtained from triplicate wells were assayed in duplicate and averaged.

### Bacterial uptake assay.

HeLa cells were seeded onto glass coverslips at 10^5^ cells per well in a 24-well tissue culture plate 24 h before infection. Cells were infected with bacteria at a calculated MOI of 50:1. After a brief centrifugation step (5 min at 100 g), the plates were incubated for 30 min at 37 °C in a 5% CO2 incubator. A double-label immunofluorescence assay was used to differentiate between extracellular and intracellular cell-associated bacteria as previously described [4]. Coverslips containing infected cells were washed with PBS and fixed in 2% paraformaldehyde for 15 min. The washed coverslips were incubated with polyclonal anti-*Yersinia* antibody SB349 (diluted 1:1000) for 40 min to stain extracellular bacteria. Washed coverslips were incubated for 40 min with FITC-conjugated goat anti-rabbit IgG diluted 1:250. After washing, cells were permeabilized with 0.2% Triton X-100 for 10 min. Coverslips were washed and incubated with SB349 (1:1,000) for 40 min to label both extracellular and intracellular bacteria. Samples were then washed and incubated for 40 min with TRITC-conjugated goat anti-rabbit IgG (1:300). All antibodies were diluted in PBS containing 3% BSA, and washes were conducted three times for 5 min with PBS containing 1% BSA. Coverslips were washed with PBS before mounting and examined by immunofluorescence microscopy. The percentage uptake was calculated as the number of [intracellular bacteria (red)/total bacteria (green and red)] × 100.

### Staining of actin cups.

The effect of Rac and Rho inhibitors on the formation of actin cups was tested in Hela cells seeded on coverslips. To inhibit Rac, the cells were treated for 6 h with NSC23766 (100μM) in 5% serum-DMEM, or 5% serum-DMEM alone. To inhibit RhoA, B and C, TATC3 (40 μg/ml) was added to the cells in serum free medium for 4 h, and control cells were incubated in serum free conditions for the same time. Hela cells were then infected for 10–15 min, washed and fixed as described above for the bacterial uptake assay. Double immunofluorescence was performed as detailed above for the bacterial uptake assay, with the addition of 50 U/ml of Rhodamine Phalloidin (Molecular Probes) together with the last secondary antibody. Images were captured with a confocal laser microscope. The percentage of bacteria (extracellular and intracellular) surrounded by an “actin halo” was calculated by counting a minimum of 150 bacteria.

### Purification of His-TAT-C3.

Plasmid pTAT–C3 (a gift from Dafna Bar Sagi, Stony Brook University, NY) was introduced into E. coli (strain BL21), and His-tagged-TAT–C3 protein were expressed by IPTG induction (1 mM IPTG, 4 h). Recombinant His-TAT–C3 was extracted from E. coli BL21 strain by sonication, and purified by fast protein liquid chromatography (FPLC), as follows. The supernatant of the cell lysate was injected onto a Hi-trap Ni-column (Pharmacia Co.). The column was washed with a 5 mM imidazole buffer solution and eluted using a gradient concentration of 1M imidazole buffer. After dialysis against PBS/0.5M NaCl, the purity of each TAT–C3 preparation was determined on polyacrylamide gels stained with Coomassie blue.

### Pull-down assay for GTP-Rho.

Cells were seeded in 10 cm dishes at 90% confluency and were left uninfected or were infected at a moi:100 for different time periods. Cells were lysed in lysis buffer (Upstate, Rho activation assay) containing 10% glycerol, and protease inhibitor (Roche). Cell lysates were clarified by centrifugation at 13,000 rpm at 4 °C for 10 min, and the supernatants were incubated with 30 μg of GST fused to the Rho binding domain of rhotekin bound to with glutathione beads, at 4 °C for 45 min. The beads were washed twice with lysis buffer and subjected to SDS-polyacrylamide gel electrophoresis on a 12% gel. Bound RhoA was detected by Western blot using a monoclonal antibody against RhoA (Santa Cruz Biotechnology).

## Supporting Information

Figure S1 Caspase-1 Inhibitor YVAD Effectively Inhibits YopB-Mediated IL-1 β Production J774A.1 cells were treated with YVAD as described in Figure 1, and left uninfected or infected with Y. pseudotuberculosis strains *yopEHJ* (YP27) or *yopEHJB* (YP29). Culture supernatants were collected from triplicate wells 6 h post infection and assayed using an IL-1 β ELISA (R&D Systems).
(18 KB PDF)

Figure S2 Effect of ToxB Treatment on Bacterial Adherence to HeLa Cells ToxB-treated or untreated cells on coverslips were infected with wild type (YP126) for 1 h and subjected to immunofluorescence to stain bacteria. The mean number of cell-associated bacteria and the standard deviation of the mean, in both conditions, were calculated by counting a minimum of 76 HeLa cells.
(20 KB PDF)

Figure S3 NFκB and MAPK Activation HeLa cells were either left uninfected or infected with strains *yopEHJ* (YP27), *yopEHJB* (YP29), *yopEHJ,yadA,inv/*p*psaABC* (YP50/pAY66), or *yopEHJB,yadA,inv/*p*psaABC*, (YP51/pAY66) for 1 h. Cells were lysed and soluble fractions of equivalent protein concentration were separated by SDS–PAGE and analyzed by immunoblotting with antibodies against IκBα, or the phosphorylated forms of ERK. Degradation of NFκB's inhibitor IκBα indirectly determines NFκB activation.
(42 KB PDF)

Figure S4 Y. pseudotuberculosis Expressing Invasin D911E Retains Most of Its Adhesive Capacity HeLa cells on coverslips were infected with Y. pseudotuberculosis strains *yopEHJ* (YP27) or *yopEHJ,yadA,invD911E* (YP50invD911E) for 1 h, and subjected to immunofluorescence. The mean number of cell-associated bacteria and the standard deviation of the mean was calculated by counting a minimum of 50 cells (A). Increasing the number of cell-associated *yadA,inv* bacteria does not ameliorate poor translocation. Y. pseudotuberculosis wild type (YP126), *yopB* mutant (YP18), *yopHJ,yadA,inv*/p*psaABC* (YP54/pAY66), or *yopHJ,yadA,invD911E* (YP54invD911E) strains were used to infect HeLa cells for 2 h at multiplicity of infection 100 or 200. Translocated YopE was analyzed by immunoblotting as described in Figure 2 (B).
(47 KB PDF)

Figure S5 Transmission Electron Microscopy Showing Binding of *Y.pseudotuberculosis* to HeLa Cells HeLa cells grown on vinyl micro slides were either untreated and infected with Y. pseudotuberculosis strains *yopEHJ* (YP27) (A), *yopEHJ,yadA,inv*/*psaABC* (YP50/pAY66) (B), or *yopEHJ,yadA,invD911E* (YP50invD911E) (C), or treated for 6 h with 100 μM NSC23766 Rac inhibitor and infected with YP27 (D). Coverslips were washed, fixed with 2.5% glutaraldehyde and processed for thin section transmission electron microscopy by the Central Microscopy Imaging Center at Stony Brook University. Digital images of the thin sections were acquired using a FEI BioTwinG2 transmission electron microscope.
(3.6 MB PDF)

Figure S6 Rac Inactivation Inhibits Formation of Nascent Phagosomes Hela cells were treated for 6 h with Rac inhibitor NSC23766 (100 μM) in 5% serum-DMEM, or with 5% serum-DMEM alone, and infected with YP27 (*yopEHJ*). The percentage of bacteria associated with actin cups was assessed 15 min after infection by double staining immunofluorescence, as described in Material and Methods. A minimum of 150 bacteria were counted (A). Fluorescence image showing actin cups. The image is a projection of several 0.8 μm Z stacks confocal microscopy pictures from HeLa cells infected with YP27 (*yopEHJ*). White arrows indicate bacteria (blue) inducing a ring of actin polymerization (red) (B).
(1.0 MB PDF)

Figure S7 Analysis of Pore Formation by Immunofluorescence in Cells Overexpressing a Dominant Negative Form of Rac Hela cells were transiently transfected with pCGTN17Rac 24h prior to infection with *yopEHJ* (YP27) strain. Infected cells were first stained with ethidium homodimer-2 (EthD2) to detect cells that have undergone pore formation. Cells were then fixed and subjected to immunofluorescence to detect T7-N17Rac using anti-T7 antibody, and anti-mouse FITC as primary and secondary antibodies, respectively (A). The percentage of cells that were permeable to the EthD-2 dye was compared among transfected and nontransfected cells. A total of 50 cells were counted (B).
(62 KB PDF)

Figure S8 Rho Inhibition Does Not Impair Bacterial Association to, or Bacterial Internalization Into HeLa Cells Hela cells were treated for 4 h with 40 μg/ml TAT-C3 in serum-free DMEM or with serum-free DMEM alone. TAT-C3-treated and untreated cells were infected with *yopEHJ* (YP27). The number of cell-associated bacteria **(A),** and internalized bacteria **(B)** was determined one h after infection by immunofluorescence, as described in Material and Methods.
(16 KB PDF)

Text S1 Supplementary Methods (23 KB DOC)

### Accession Numbers

The GenBank (http://www.ncbi.nlm.nih.gov/Genbank/index.html) accession number for invasin is M17448.

## References

[ppat-0040003-b001] Cornelis GR (2000). Molecular and cell biology aspects of plague. Proc Natl Acad Sci U S A.

[ppat-0040003-b002] Viboud GI, Bliska JB (2005). *Yersinia* outer proteins: role in modulation of host cell signaling responses and pathogenesis. Annu Rev Microbiol.

[ppat-0040003-b003] Barbieri JT, Riese MJ, Aktories K (2002). Bacterial toxins that modify the actin cytoskeleton. Annu Rev Cell Dev Biol.

[ppat-0040003-b004] Black DS, Bliska JB (2000). The RhoGAP activity of the Yersinia pseudotuberculosis cytotoxin YopE is required for antiphagocytic function and virulence. Mol Microbiol.

[ppat-0040003-b005] Von Pawel-Rammingen U, Telepnev MV, Schmidt G, Aktories K, Wolf-Watz H (2000). GAP activity of the yersinia YopE cytotoxin specifically targets the rho pathway: a mechanism for disruption of actin microfilament structure. Mol Microbiol.

[ppat-0040003-b006] Shao F, Dixon JE (2003). YopT is a cysteine protease cleaving Rho family GTPases. Adv Exp Med Biol.

[ppat-0040003-b007] Håkansson S, Schesser K, Persson C, Galyov EE, Rosqvist R (1996). The YopB protein of Yersinia pseudotuberculosis is essential for the translocation of Yop effector proteins across the target cell plasma membrane and displays a contact-dependent membrane disrupting activity. EMBO J.

[ppat-0040003-b008] Neyt C, Cornelis G (1999). Insertion of a Yop translocation pore into the macrophage plasma membrane by Yersinia enterocolitica: requirement for translocators YopB and YopD, but not LcrG. Mol Microbiol.

[ppat-0040003-b009] Tardy F, Homble F, Neyt C, Wattiez R, Cornelis GR (1999). Yersinia enterocolitica type III secretion-translocation system: channel formation by secreted Yops. Embo J.

[ppat-0040003-b010] Mueller CA, Broz P, Muller SA, Ringler P, Erne-Brand F (2005). The V-antigen of *Yersinia* forms a distinct structure at the tip of injectisome needles. Science.

[ppat-0040003-b011] Broz P, Mueller CA, Muller SA, Philippsen A, Sorg I (2007). Function and molecular architecture of the *Yersinia* injectisome tip complex. Mol Microbiol.

[ppat-0040003-b012] Eitel J, Dersch P (2002). The YadA protein of Yersinia pseudotuberculosis mediates high-efficiency uptake into human cells under environmental conditions in which invasin is repressed. Infect Immun.

[ppat-0040003-b013] Payne D, Tatham D, Williamson ED, Titball RW (1998). The pH 6 antigen of Yersinia pestis binds to β1-linked galactosyl residues in glycosphingolipids. Infect Immun.

[ppat-0040003-b014] Owen KA, Thomas KS, Bouton AH (2007). The differential expression of Yersinia pseudotuberculosis adhesins determines the requirement for FAK and/or Pyk2 during bacterial phagocytosis by macrophages. Cell Microbiol.

[ppat-0040003-b015] Wong KW, Isberg RR (2005). Emerging views on integrin signaling via Rac1 during invasin-promoted bacterial uptake. Curr Opin Microbiol.

[ppat-0040003-b016] Viboud GI, So SS, Ryndak MB, Bliska JB (2003). Proinflammatory signalling stimulated by the type III translocation factor YopB is counteracted by multiple effectors in epithelial cells infected with Yersinia pseudotuberculosis. Mol Microbiol.

[ppat-0040003-b017] Viboud GI, Mejia E, Bliska JB (2006). Comparison of YopE and YopT activities in counteracting host signalling responses to Yersinia pseudotuberculosis infection. Cell Microbiol.

[ppat-0040003-b018] Viboud GI, Bliska JB (2001). A bacterial type III secretion system inhibits actin polymerization to prevent pore formation in host cell membranes. Embo J.

[ppat-0040003-b019] Marenne MN, Journet L, Mota LJ, Cornelis GR (2003). Genetic analysis of the formation of the Ysc-Yop translocation pore in macrophages by Yersinia enterocolitica: role of LcrV, YscF and YopN. Microb Pathog.

[ppat-0040003-b020] Fink SL, Cookson BT (2005). Apoptosis, pyroptosis, and necrosis: mechanistic description of dead and dying eukaryotic cells. Infect Immun.

[ppat-0040003-b021] Fink SL, Cookson BT (2006). Caspase-1-dependent pore formation during pyroptosis leads to osmotic lysis of infected host macrophages. Cell Microbiol.

[ppat-0040003-b022] Viboud GI, Bliska JB (2002). Measurement of pore formation by contact-dependent type III protein secretion systems. Methods Enzymol.

[ppat-0040003-b023] Schotte P, Denecker G, Van Den Broeke A, Vandenabeele P, Cornelis GR (2004). Targeting Rac1 by the *Yersinia* effector protein YopE inhibits caspase-1-mediated maturation and release of interleukin-1beta. J Biol Chem.

[ppat-0040003-b024] Aili M, Isaksson EL, Hallberg B, Wolf-Watz H, Rosqvist R (2006). Functional analysis of the YopE GTPase-activating protein (GAP) activity of Yersinia pseudotuberculosis. Cell Microbiol.

[ppat-0040003-b025] Aili M, Isaksson EL, Carlsson SE, Wolf-Watz H, Rosqvist R (2007). Regulation of *Yersinia* Yop-effector delivery by translocated YopE. Int J Med Microbiol.

[ppat-0040003-b026] Wong KW, Isberg RR (2005). Yersinia pseudotuberculosis spatially controls activation and misregulation of host cell Rac1. PLoS Pathog.

[ppat-0040003-b027] Yang Y, Isberg RR (1997). Transcriptional regulation of the Yersinia pseudotuberculosis pH6 antigen adhesin by two envelope-associated components. Mol Microbiol.

[ppat-0040003-b028] Marra A, Isberg RR (1997). Invasin-dependent and invasin-independent pathways for translocation of Yersinia pseudotuberculosis across the Peyer's patch intestinal epithelium. Infect Immun.

[ppat-0040003-b029] Gao Y, Dickerson JB, Guo F, Zheng J, Zheng Y (2004). Rational design and characterization of a Rac GTPase-specific small molecule inhibitor. Proc Natl Acad Sci U S A.

[ppat-0040003-b030] Alrutz MA, Srivastava A, Wong KW, D'Souza-Schorey C, Tang M (2001). Efficient uptake of Yersinia pseudotuberculosis via integrin receptors involves a Rac1-Arp 2/3 pathway that bypasses N-WASP function. Mol Microbiol.

[ppat-0040003-b031] Zaharik ML, Gruenheid S, Perrin AJ, Finlay BB (2002). Delivery of dangerous goods: type III secretion in enteric pathogens. Int J Med Microbiol.

[ppat-0040003-b032] Olsson J, Edqvist PJ, Broms JE, Forsberg A, Wolf-Watz H (2004). The YopD translocator of Yersinia pseudotuberculosis is a multifunctional protein comprised of discrete domains. J Bacteriol.

[ppat-0040003-b033] Shin H, Cornelis GR (2007). Type III secretion translocation pores of Yersinia enterocolitica trigger maturation and release of pro-inflammatory IL-1 β. Cellular Microbiology.

[ppat-0040003-b034] Sory M-P, Boland A, Lambermount I, Cornelis G (1995). Identification of the YopE and YopH domains required for secretion and internalization into the cytosol of macrophages, using the *cyaA* gene fusion approach. Proc Natl Acad Sci USA.

[ppat-0040003-b035] Rosqvist R, Hakansson S, Forsberg A, Wolf-Watz H (1995). Functional conservation of the secretion and translocation machinery for virulence proteins of yersiniae, salmonellae and shigellae. EMBO J.

[ppat-0040003-b036] Mota LJ, Journet L, Sorg I, Agrain C, Cornelis GR (2005). Bacterial injectisomes: needle length does matter. Science.

[ppat-0040003-b037] Werner E, Kheradmand F, Isberg RR, Werb Z (2001). Phagocytosis mediated by *Yersinia* invasin induces collagenase-1 expression in rabbit synovial fibroblasts through a proinflammatory cascade. J Cell Sci.

[ppat-0040003-b038] Watarai M, Kamata Y, Kozaki S, Sasakawa C (1997). rho, a small GTP-binding protein, is essential for *Shigella* invasion of epithelial cells. J Exp Med.

[ppat-0040003-b039] Watarai M, Funato S, Sasakawa C (1996). Interaction of Ipa proteins of Shigella flexneri with alpha5beta1 integrin promotes entry of the bacteria into mammalian cells. J Exp Med.

[ppat-0040003-b040] Palazzo AF, Eng CH, Schlaepfer DD, Marcantonio EE, Gundersen GG (2004). Localized stabilization of microtubules by integrin- and FAK-facilitated Rho signaling. Science.

[ppat-0040003-b041] Hayward RD, Cain RJ, McGhie EJ, Phillips N, Garner MJ (2005). Cholesterol binding by the bacterial type III translocon is essential for virulence effector delivery into mammalian cells. Mol Microbiol.

[ppat-0040003-b042] Hao S, August A (2005). Actin depolymerization transduces the strength of B-cell receptor stimulation. Mol Biol Cell.

[ppat-0040003-b043] Villalba M, Bi K, Rodriguez F, Tanaka Y, Schoenberger S (2001). Vav1/Rac-dependent actin cytoskeleton reorganization is required for lipid raft clustering in T cells. J Cell Biol.

[ppat-0040003-b044] Fassett MS, Davis DM, Valter MM, Cohen GB, Strominger JL (2001). Signaling at the inhibitory natural killer cell immune synapse regulates lipid raft polarization but not class I MHC clustering. Proc Natl Acad Sci U S A.

[ppat-0040003-b045] Enninga J, Mounier J, Sansonetti P, Tran Van Nhieu G (2005). Secretion of type III effectors into host cells in real time. Nat Methods.

[ppat-0040003-b046] Schlumberger MC, Muller AJ, Ehrbar K, Winnen B, Duss I (2005). Real-time imaging of type III secretion: *Salmonella* SipA injection into host cells. Proc Natl Acad Sci U S A.

[ppat-0040003-b047] Zauberman A, Cohen S, Mamroud E, Flashner Y, Tidhar A (2006). Interaction of Yersinia pestis with macrophages: limitations in YopJ-dependent apoptosis. Infect Immun.

[ppat-0040003-b048] Hueck CJ (1998). Type III protein secretion systems in bacterial pathogens of animals and plants. Microbiol Mol Biol Rev.

[ppat-0040003-b049] Bolin I, Norlander L, Wolf-Watz H (1982). Temperature-inducible outer membrane protein of Yersinia pseudotuberculosis and Yersinia enterocolitica is associated with the virulence plasmid. Infect Immun.

[ppat-0040003-b050] Viboud GI, Mejia E, Bliska JB (2006). Comparison of YopE and YopT activities in counteracting host signalling responses to Yersinia pseudotuberculosis infection. Cellular Microbiology.

[ppat-0040003-b051] Palmer LE, Hobbie S, Galan JE, Bliska JB (1998). YopJ of Yersinia pseudotuberculosis is required for the inhibition of macrophage TNFα production and the downregulation of the MAP kinases p38 and JNK. Mol Microbiol.

[ppat-0040003-b052] Bliska JB, Copass MC, Falkow S (1993). The Yersinia pseudotuberculosis adhesin YadA mediates intimate bacterial attachment to and entry into HEp-2 cells. Infect Immun.

[ppat-0040003-b053] Palmer LE, Pancetti AR, Greenberg S, Bliska JB (1999). YopJ of Yersinia spp. is sufficient to cause downregulation of multiple mitogen activated protein kinases in eukaryotic cells. Infect Immun.

[ppat-0040003-b054] Simonet M, Falkow S (1992). Invasin expression in Yersinia pseudotuberculosis. Infect Immun.

[ppat-0040003-b055] Joneson T, White MA, Wigler MH, Bar-Sagi D (1996). Stimulation of membrane ruffling and MAP kinase activation by distinct effectors of RAS. Science.

[ppat-0040003-b056] Nimnual AS, Yatsula BA, Bar-Sagi D (1998). Coupling of Ras and Rac guanosine triphosphatases through the Ras exchanger Sos. Science.

