# Supplementary material for: Yersinia Controls Type III Effector Delivery into Host Cells by Modulating Rho Activity
Source: PLoS Pathog. 2008 Jan 11;4(1):e3. doi: 10.1371/journal.ppat.0040003 (PMC2186360; doi:10.1371/journal.ppat.0040003)
Supplement: Supplementary file 1 [file ppat.0040003.sg001.pdf]

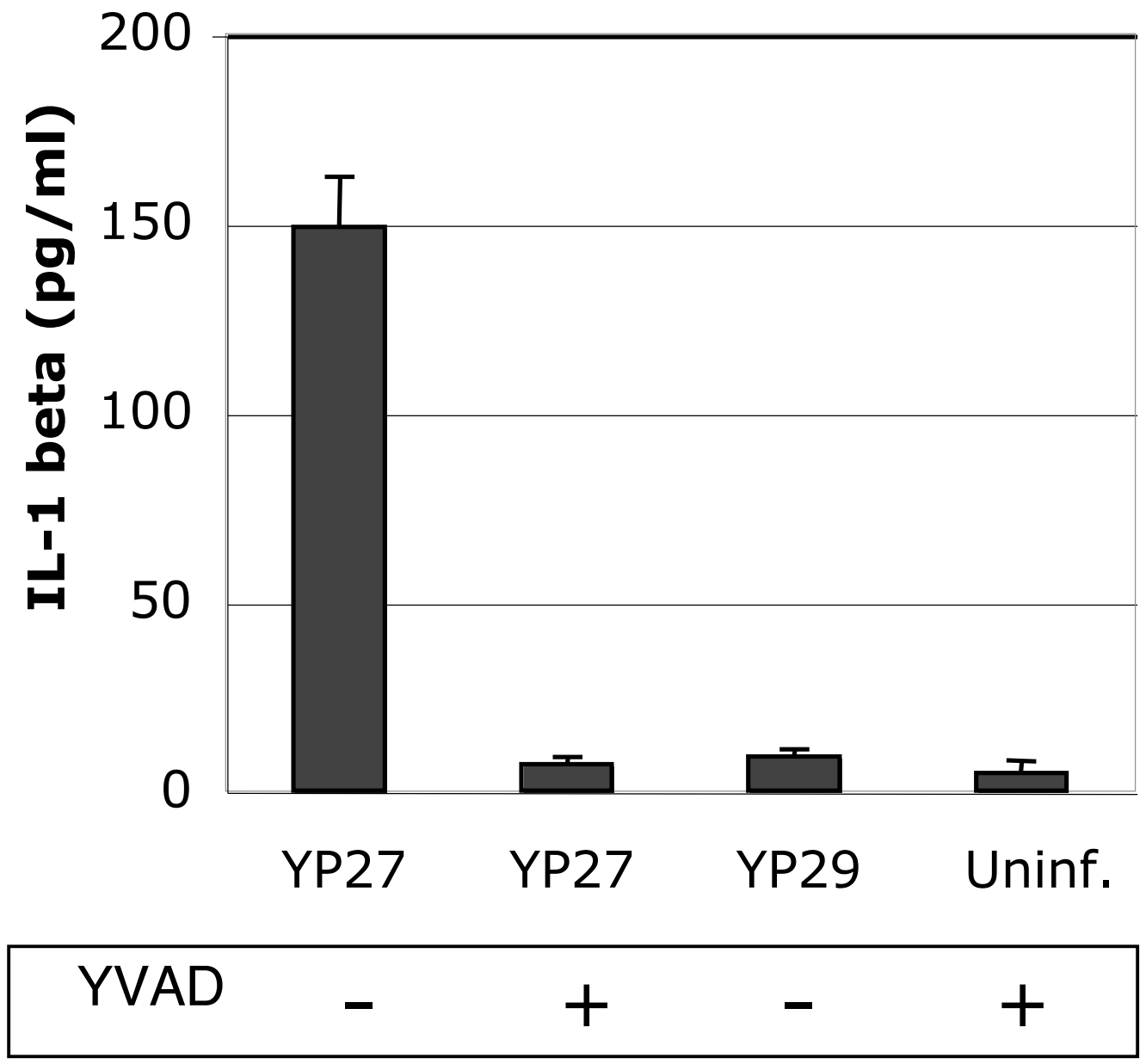
200
150
100
50
0
IL-1 beta (pg/ml)
YP27
YP27
YP29
Uninf.
YVAD
-
+
-
+
