# Supplementary material for: Yersinia Controls Type III Effector Delivery into Host Cells by Modulating Rho Activity
Source: PLoS Pathog. 2008 Jan 11;4(1):e3. doi: 10.1371/journal.ppat.0040003 (PMC2186360; doi:10.1371/journal.ppat.0040003)

A

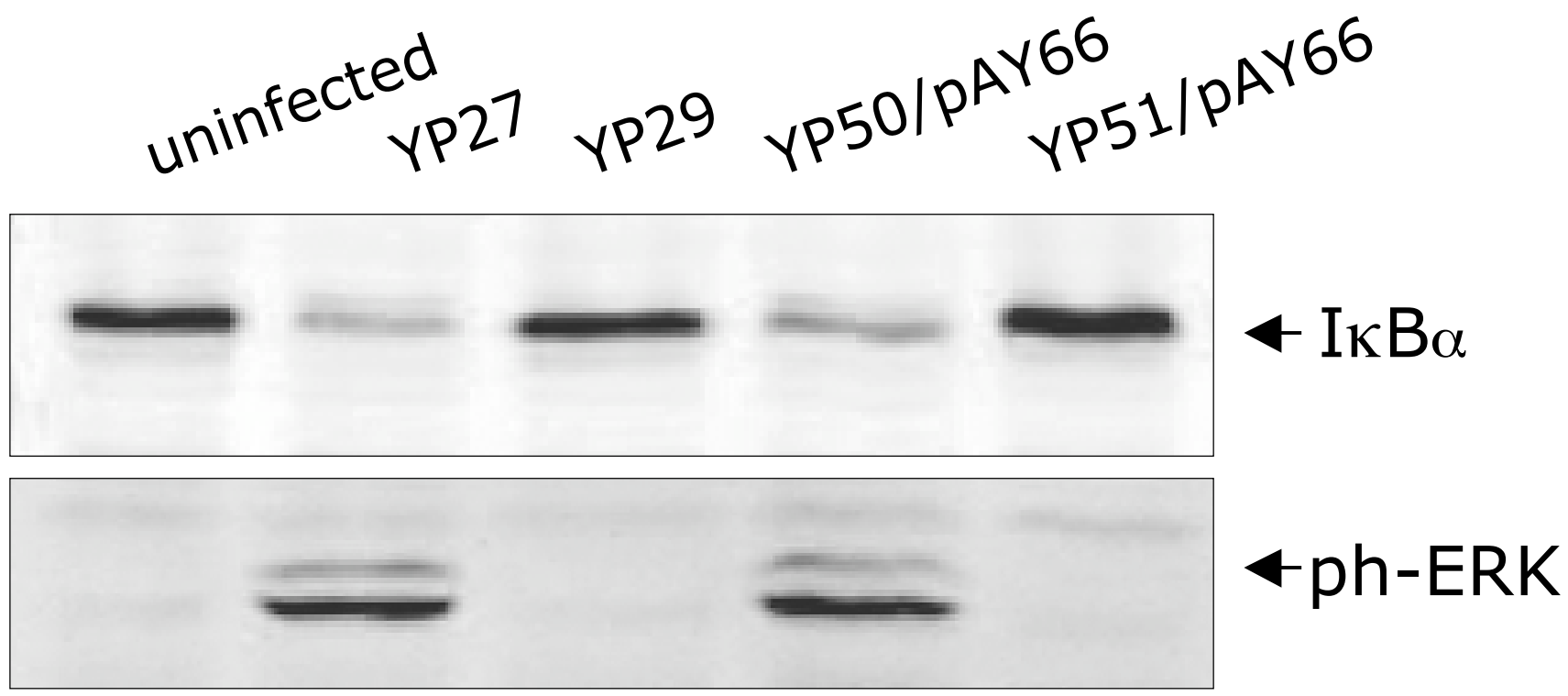

Supplement: Supplementary file 3 [file ppat.0040003.sg003.pdf]
