# Supplementary material for: Yersinia Controls Type III Effector Delivery into Host Cells by Modulating Rho Activity
Source: PLoS Pathog. 2008 Jan 11;4(1):e3. doi: 10.1371/journal.ppat.0040003 (PMC2186360; doi:10.1371/journal.ppat.0040003)
Supplement: Supplementary file 4 [file ppat.0040003.sg004.pdf]

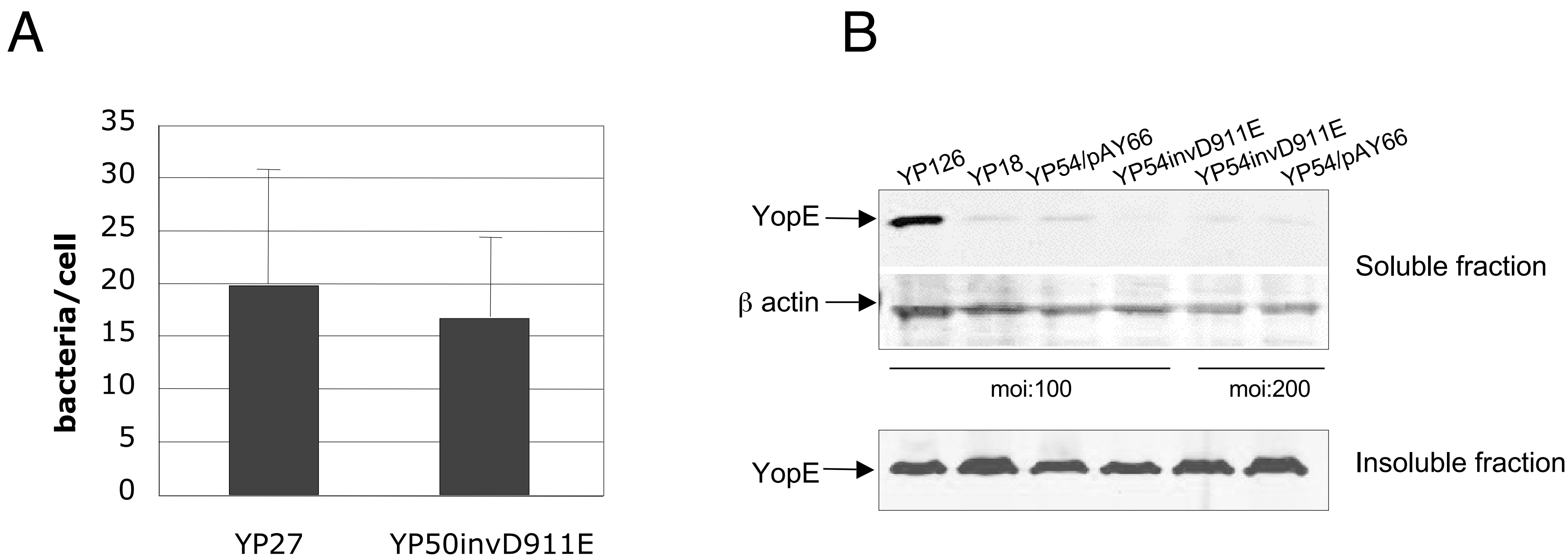

A
35
30
25
20
15
10
5
0
bacteria/cell
YP27
YP50invD911E
B
YP126
YP18
YP54/pAY66
YP54invD911E
YP54invD911E
YP54/pAY66
YopE
β actin
Soluble fraction
moi:100
moi:200
YopE
Insoluble fraction
