# Supplementary material for: Yersinia Controls Type III Effector Delivery into Host Cells by Modulating Rho Activity
Source: PLoS Pathog. 2008 Jan 11;4(1):e3. doi: 10.1371/journal.ppat.0040003 (PMC2186360; doi:10.1371/journal.ppat.0040003)
Supplement: Supplementary file 5 [file ppat.0040003.sg005.pdf]

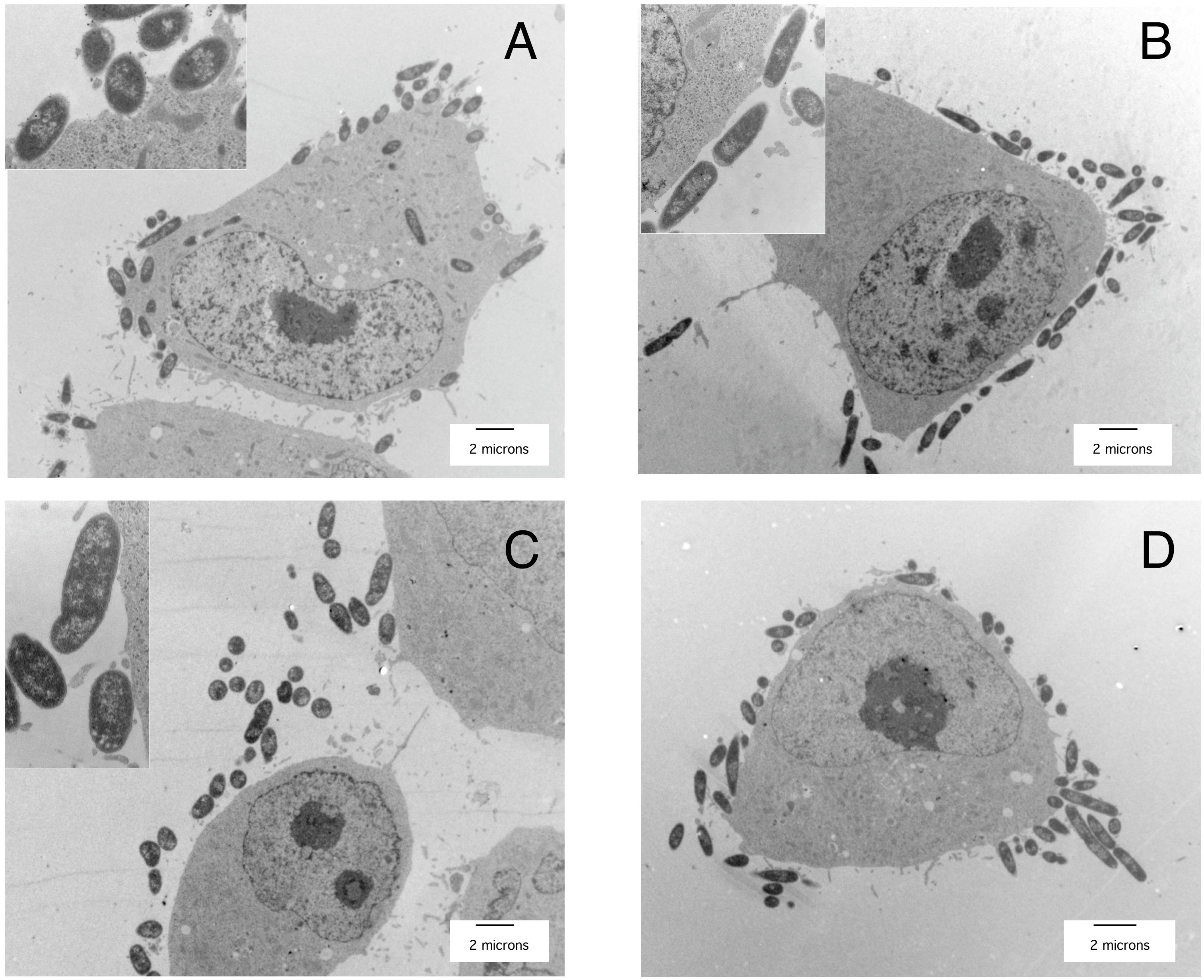
A
2 microns
B
2 microns
C
2 microns
D
2 microns
