# Supplementary material for: Yersinia Controls Type III Effector Delivery into Host Cells by Modulating Rho Activity
Source: PLoS Pathog. 2008 Jan 11;4(1):e3. doi: 10.1371/journal.ppat.0040003 (PMC2186360; doi:10.1371/journal.ppat.0040003)
Supplement: Supplementary file 7 [file ppat.0040003.sg007.pdf]

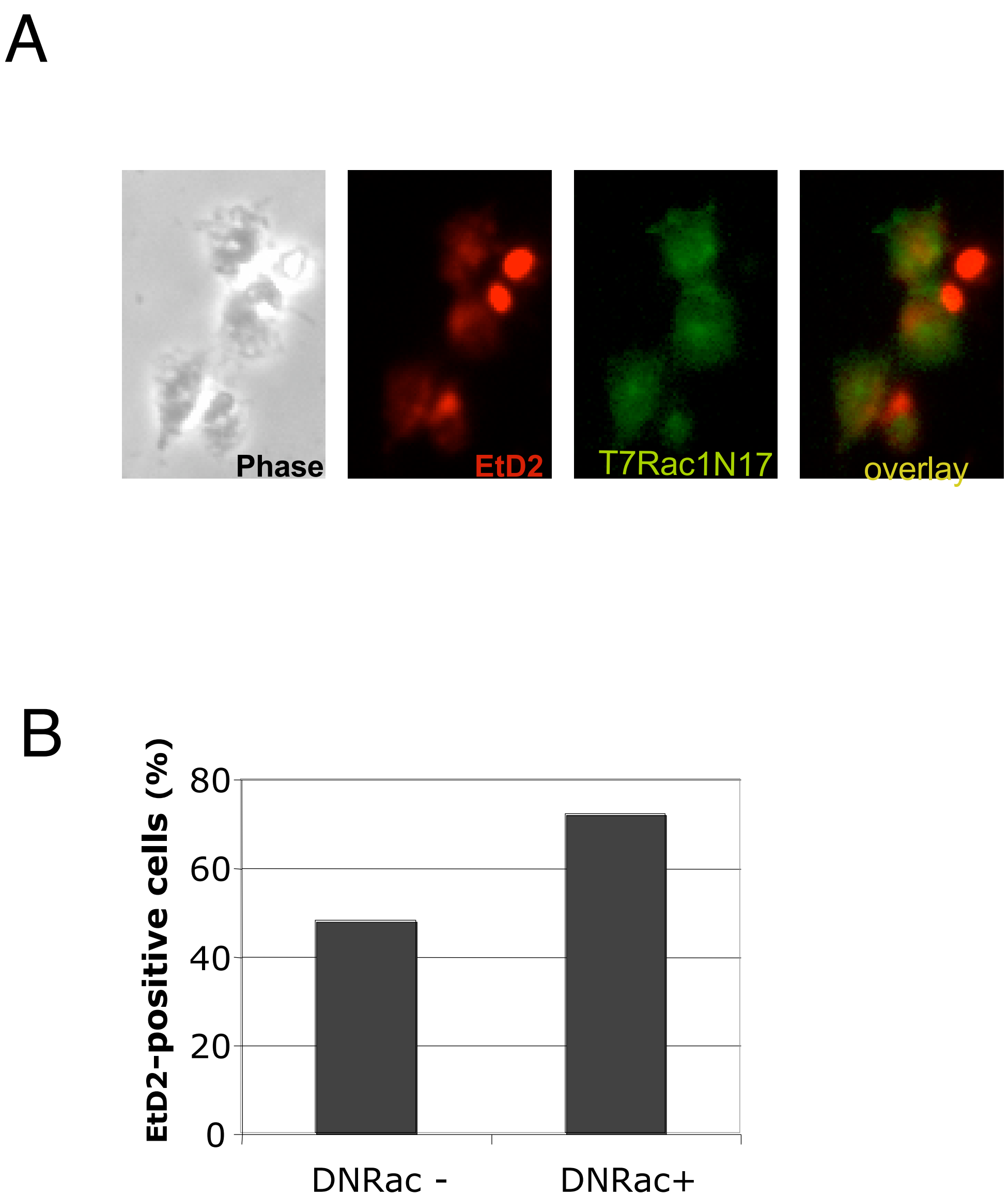
A
Phase
EtD2
T7Rac1N17
overlay
B
EtD2-positive cells (%)
80
60
40
20
0
DNRac -
DNRac+
