# Supplementary material for: Yersinia Controls Type III Effector Delivery into Host Cells by Modulating Rho Activity
Source: PLoS Pathog. 2008 Jan 11;4(1):e3. doi: 10.1371/journal.ppat.0040003 (PMC2186360; doi:10.1371/journal.ppat.0040003)
Supplement: Supplementary file 8 [file ppat.0040003.sg008.pdf]

A

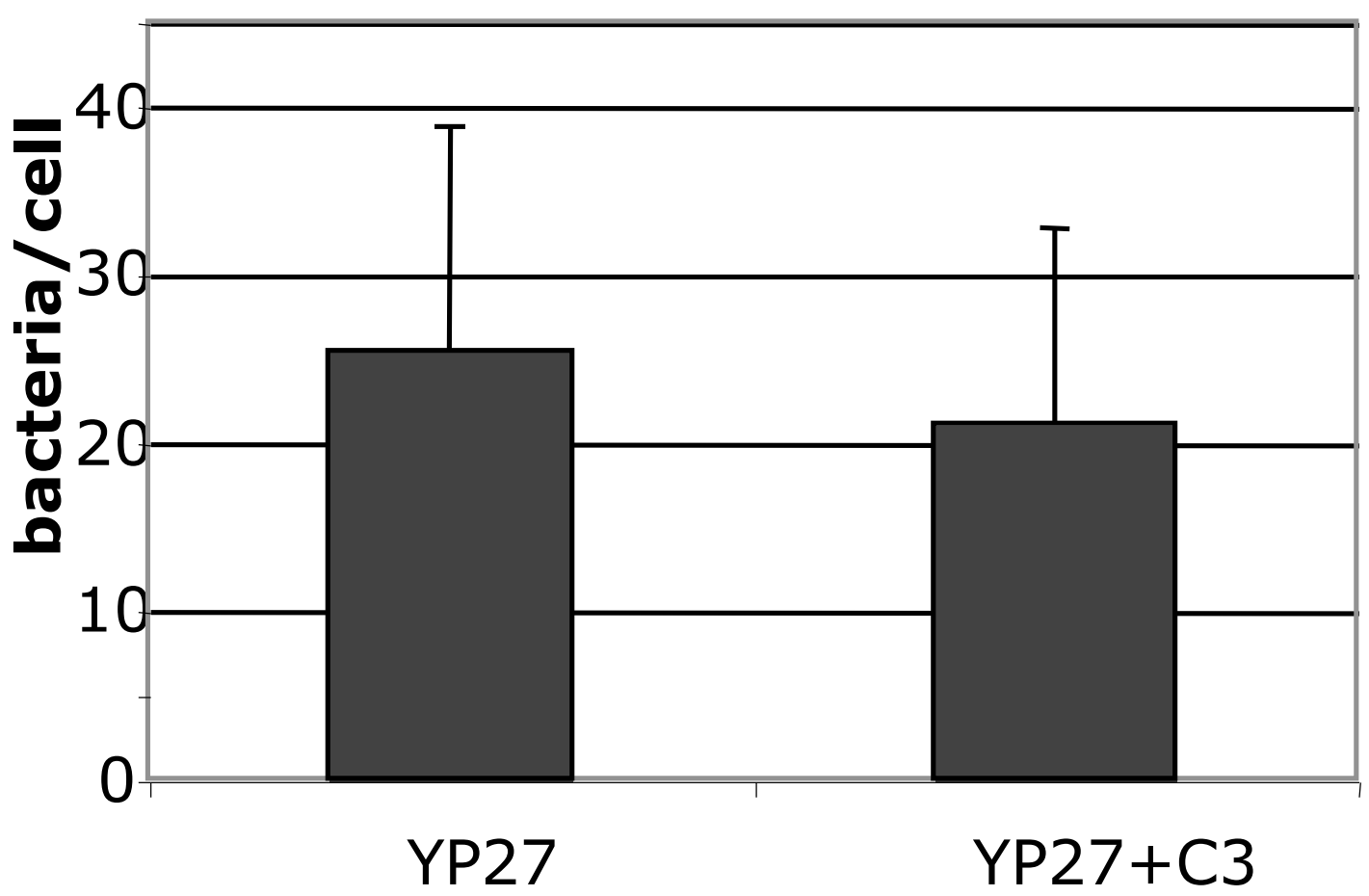
bacteria/cell
40
30
20
10
0
YP27
YP27+C3

B

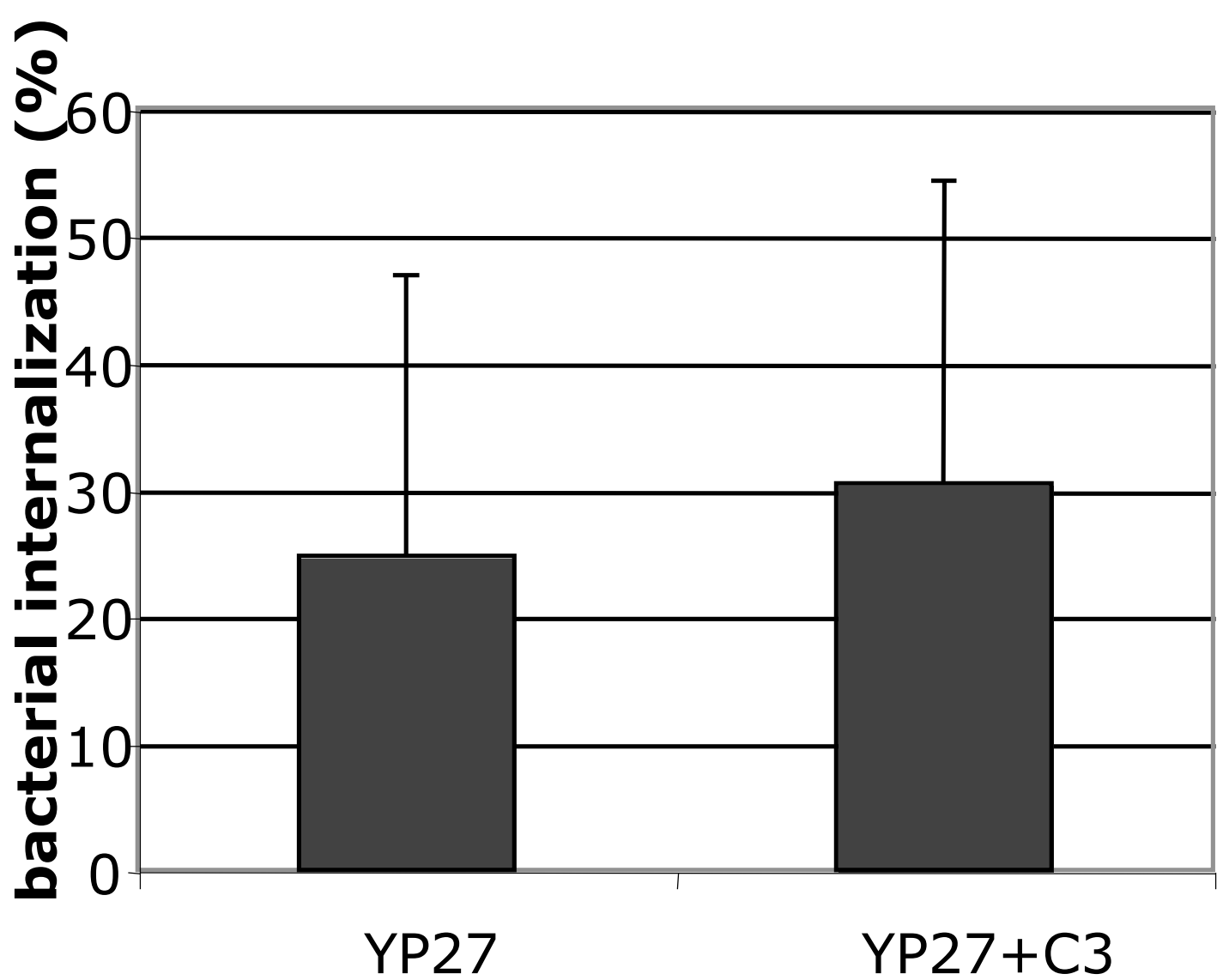
bacterial internalization (%)
60
50
40
30
20
10
0
YP27
YP27+C3
