# Supplementary material for: Yersinia Controls Type III Effector Delivery into Host Cells by Modulating Rho Activity
Source: PLoS Pathog. 2008 Jan 11;4(1):e3. doi: 10.1371/journal.ppat.0040003 (PMC2186360; doi:10.1371/journal.ppat.0040003)
Supplement: Supplementary file 9 [file ppat.0040003.sd001.doc]

**Text S1**

**IL-1**β **ELISA.** J774A1 cells cultured in 24 well plates in DMEM Glutamax (Gibco) with 10% fetal calf serum and 1% sodium pyruvate, were infected as described in Schotte *et al.* [23] in the presence or absence of YVAD. Briefly, culture supernatants from triplicate wells were collected 6h post infection and assayed using an IL-1 β ELISA (R&D Systems) following instructions from the manufacturers.

**Electron microscopy.** Cells were grown on vinyl Rinzl film, infected for 1 hour at a moi of 100, washed, and fixed in 2.5% EM grade glutaraldehyde in PBS.  Fixed cells were then processed using standard EM techniques.  Briefly, samples were then placed in 2% osmium tetroxidein PBS, dehydrated in a graded series of ethyl alcohol and embedded in Durcupan resin. Areas of interest were blocked and 80nm ultrathin sections were cut with a Reichert-Jung UltracutE ultramicrotome and placed on formvar coated slot copper grids.  Sections were then counterstained with uranyl acetate and lead citrate and viewed with a FEI Tecanai BioTwinG^2^ transmission electron microscope.  Digital images were aquired with an AMT XR-60 CCD digital camera system and compiled using Adobe Photoshop.

**MAPK and IκB**α **Immunoblotting.** HeLa cells seeded in 60mm tissue culture dishes were infected for 1 hour washed and lysed in 1% Triton X-100 buffer. Immunoblotting was performed using polyclonal antibodies against phospho ERK (Cell Signaling) according to the procedures of the manufacturer. Activation of NFκB was indirectly determined by assessing the degradation of its inhibitor IκBα in the lysates of infected cells.

**Adhesion of *Yersinia* to HeLa cells.** Hela cells cultured on coverslips were infected as for the bacterial uptake assay. After 1 hour infection, cells were washed with PBS, fixed and processed by immunofluorescence with anti-yersinia antibody as described for the bacteria uptake assay. The membrane of the HeLa cell was stained with Texas Red wheat germ agglutinin. The percentage of adherent bacteria was calculated by counting a minimum of 50 infected cells.
